# FNDC5/Irisin-dependent renoprotection of resistance training in myocardial infarction–induced Type 2 cardiorenal syndrome

**DOI:** 10.1371/journal.pone.0342468

**Published:** 2026-02-20

**Authors:** Weiyu Fu, Jun Lin, Wenqian Lin, Kai Zeng

**Affiliations:** 1 Department of Anesthesiology, The First Affiliated Hospital, Fujian Medical University, Fuzhou, China; 2 Department of Anesthesiology, National Regional Medical Center, Binhai Campus of the First Affiliated Hospital, Fujian Medical University, Fuzhou, China; 3 Anesthesiology Research Institute, The First Affiliated Hospital, Fujian Medical University, Fuzhou, China; University of Diyala College of Medicine, IRAQ

## Abstract

Type 2 cardiorenal syndrome (CRS), driven by chronic myocardial infarction (MI), is characterized by renal fibrosis and oxidative stress, yet underlying mechanisms and therapies are poorly defined. This study investigated whether resistance training protects against MI-induced renal injury via the FNDC5/Irisin axis. Male wild-type (WT) and global Fndc5 knockout (KO) mice were subjected to MI or sham surgery and then allocated to sedentary or ladder-climbing resistance training groups for 4 weeks (n = 8 per group). An in vitro model was established using H_2_O_2_-stimulated human renal tubular (HKC) cells. We found that resistance training upregulated renal FNDC5 expression, lowered serum creatinine and blood urea nitrogen levels, and attenuated tubular injury in WT mice with CRS, but these benefits were markedly blunted in KO mice. Training reduced renal malondialdehyde content, enhanced superoxide dismutase 1/2 expression, and decreased collagen deposition alongside downregulation of fibrotic markers (Collagen-I/III, α-SMA). These improvements were associated with suppressed activation of the renal TGF-β1/Smad2/3 pathway in WT but not in KO mice. In HKC cells, recombinant Irisin and the AMPK agonist AICAR mitigated H_2_O_2_-induced oxidative stress, fibrotic protein expression, and Smad2/3 phosphorylation. We conclude that resistance exercise ameliorates renal oxidative stress and fibrosis in Type 2 CRS, effects that are substantially mediated by, but not exclusively dependent on, the FNDC5/Irisin axis. Our work highlights FNDC5/Irisin as a key amplifier of exercise-induced renoprotection and supports the therapeutic potential of resistance training in cardiorenal syndrome.

## 1. Introduction

Heart failure (HF) is a major cause of mortality among individuals with non-communicable diseases worldwide [1]. HF frequently leads to complications involving distant organs, with renal impairment being particularly common [2]. Clinical studies have reported that patients with chronic HF often exhibit signs of renal insufficiency or dysfunction [3]. Type 2 cardiorenal syndrome (Type 2 CRS) refers to a clinical condition characterized by structural and functional renal impairment resulting from prolonged myocardial ischemia and hypoxia due to myocardial infarction (MI) or chronic HF [4,5]. Cardiorenal syndromes are classified into five subtypes based on the primary organ of insult and the chronicity of the condition. Type 1 CRS involves acute cardiac dysfunction (e.g., acute heart failure) leading to acute kidney injury, whereas Type 2 CRS is defined by chronic cardiac dysfunction (e.g., chronic heart failure) causing progressive chronic kidney disease. In this study, we focused on Type 2 CRS induced by chronic MI, as it represents a prevalent and therapeutically challenging clinical trajectory where long-term, modifiable interventions such as exercise are of particular relevance. Evidence suggests that HF patients with concomitant renal injury exhibit significantly worse cardiac dysfunction and higher mortality risk compared to those without renal involvement [6,7]. Improving renal function has been shown to enhance cardiac performance and survival outcomes in HF patients, thereby reducing disease-related mortality [8,9]. Thus, identifying effective strategies to prevent or mitigate renal structural damage and functional decline in Type 2 CRS is of critical importance for improving prognosis.

Following MI, weakened myocardial contractility and reduced cardiac output elevate peripheral vascular resistance, resulting in sustained renal ischemia and hypoxia. This, in turn, intensifies oxidative stress and inflammatory responses in renal tissues, promotes collagen deposition in the renal interstitium, and ultimately leads to renal interstitial fibrosis (RIF) and renal dysfunction [10]. Exercise is recognized as a key intervention for the prevention and rehabilitation of chronic diseases [11]. Studies have shown that aerobic exercise can ameliorate renal impairment in Type 2 CRS [12], and resistance exercise can significantly inhibit renal fibrosis and improve kidney function in animal models of chronic kidney disease [13]. However, whether resistance exercise can effectively suppress renal interstitial collagen deposition in the context of Type 2 CRS remains unclear.

Irisin, a newly identified exercise-induced myokine, has been shown to exert a range of biological effects, including regulation of energy metabolism and inhibition of oxidative stress, fibrosis, and apoptosis [14,15]. Exercise training activates peroxisome proliferator-activated receptor gamma coactivator 1-alpha (PGC-1α), which induces the expression of downstream FNDC5; this precursor protein undergoes cleavage to generate circulating Irisin [16]. Irisin exerts protective effects in multiple organs, including the heart, skeletal muscle, kidney, and brain [17,18]. In mice, Irisin administration alleviates angiotensin II (Ang II)-induced myocardial fibrosis [19]. Aerobic exercise has been shown to upregulate renal Irisin expression in MI mice, thereby reducing renal damage [12]. Nevertheless, it remains unknown whether resistance exercise can stimulate endogenous Irisin expression and consequently improve renal injury in Type 2 CRS, as well as the underlying mechanisms involved.

To address this question, the present study employed both wild-type (WT) and global FNDC5/Irisin knockout mice to establish a MI-induced Type 2 CRS model combined with a resistance exercise intervention. Additionally, an in vitro fibrosis model was established by exposing human embryonic kidney tubular epithelial (HKC) cells to H_2_O_2_-induced oxidative stress. The aim was to investigate the role and mechanism of Irisin in mediating the effects of resistance exercise on renal interstitial collagen deposition in Type 2 CRS. This study provides experimental evidence to support the use of exercise rehabilitation strategies for patients with cardiorenal syndrome.

## 2. Materials and methods

### 2.1 Experimental animals, model establishment, and exercise protocol

All animal experiments were conducted in accordance with the guidelines set by the Animal Care and Use Committee of Qingdao Harwars Biology Group Ltd. The study protocol was approved by the committee under protocol number AUP-QY-C-S-2024–033.

Eight-week-old healthy male C57BL/6J mice (purchased from the Animal Experiment Center of Xi’an Jiaotong University) and global FNDC5/Irisin knockout mice (customized by Cyagen Biosciences Inc.) were housed under standard laboratory conditions. FNDC5 knockout (KO) mice were generated on a C57BL/6J background via CRISPR/Cas9-mediated deletion of exons 2–3 of the FNDC5 gene, resulting in a null allele. WT and KO mice were not littermates but were age-matched and derived from the same inbred colony to minimize background variability. All the mice were housed in individually ventilated cages (4 mice per cage; 32 × 20 × 15 cm), with a controlled environment (22 ± 2^o^C, 55% ± 10% humidity) and a 12-hour light/dark cycle (lights on from 7:00 a.m. to 7:00 p.m.). Food and water were provided ad libitum. All animal procedures complied with the Guidelines for the Care and Use of Laboratory Animals.

A total of 24 wild-type (WT) mice were randomly divided into three groups: sham-operated group (S), myocardial infarction group (MI), and myocardial infarction plus resistance exercise group (MR). Animals were randomly assigned to experimental groups using a computer-generated block randomization method, stratified by body weight. Littermate distribution across groups was not applicable due to the use of separately bred WT and KO lines. Investigators were blinded to group allocation during data collection and analysis. Similarly, 24 FNDC5/Irisin knockout mice were randomly assigned to the knockout sham group (KS), knockout MI group (KMI), and knockout MI plus resistance exercise group (KMR). Myocardial infarction (MI) was induced by permanent ligation of the left anterior descending coronary artery under isoflurane inhalation anesthesia. Echocardiography was performed postoperatively to confirm the success and consistency of modeling. Only mice with comparable echocardiographic outcomes were randomly assigned to experimental groups. The sham groups (S and KS) underwent thoracotomy and suture placement without coronary ligation to exclude surgical stress effects.

Resistance training was conducted based on previously described protocols [20,21]. Starting one week after surgery, mice in the MR and KMR groups underwent ladder-climbing resistance training. The climbing ladder was 1.1 meters high with 1-cm grid spacing and an 85° incline. Mice were first subjected to 5 days of adaptive training without additional load. Formal training then proceeded for 4 weeks with progressive loading. The load was increased daily by 10% of body weight until reaching 75%, which was maintained until the end of the intervention. Each training session consisted of 8 sets of 3 climbs per set, with 1-minute rest between sets, 5 days per week for 4 weeks. Exercise performance was monitored throughout the intervention. Both WT and FNDC5 KO mice were able to complete the prescribed ladder-climbing sessions under the same progressive load regimen. KO mice occasionally required slightly longer to complete individual climbs, particularly during the adaptation phase, but overall compliance with the protocol was comparable between groups. Meanwhile, the test was conducted in compliance with the ARRIVE (Animal Research: Reporting In Vivo Experiments) guidelines to ensure transparency in the reporting of animal experiments. Furthermore, only male mice were used in this study to avoid potential confounding effects of the estrous cycle on cardiovascular and renal physiology, particularly in models involving oxidative stress and fibrosis. While this limits generalizability to females, male-only cohorts are commonly employed in preclinical CRS models to reduce variability in outcome measures. Future studies including both sexes are warranted.

### 2.2 Humane endpoints and animal monitoring

Humane endpoints were strictly defined and implemented to minimize animal suffering. The criteria for euthanasia included: 1) severe reduction in spontaneous activity and unresponsiveness to external stimuli; 2) labored or dyspneic breathing; 3) inability to access food or water autonomously, leading to a > 20% loss of baseline body weight; or 4) signs of severe and persistent distress (e.g., self-mutilation, hunched posture). Any animal meeting these endpoint criteria was euthanized immediately by an overdose of isoflurane inhalation. No animals died prior to reaching the humane endpoints during this study. The total duration of the experiment was 5 weeks (1-week postoperative recovery followed by 4 weeks of exercise intervention). The health and behavior of all mice were monitored every 4 hours for the first 24 hours post-surgery and twice daily thereafter. To alleviate postoperative pain, all mice undergoing thoracotomy received analgesia with subcutaneous bupivacaine (0.25%, 1 mg/kg) during surgery and for 24 hours postoperatively. All research staff were trained in laboratory animal care, handling, and the analgesic protocol.

### 2.3 HKC cell culture and interventions

Human embryonic kidney tubular epithelial (HKC) cells were cultured in F12/DMEM (1:1) medium under standard conditions (37°C, 5% CO_2_, Thermo Incubator). Cells between passages 3 and 7 were used for experiments. Oxidative stress-induced fibrosis was modeled by exposing cells to 100 μmol/L H_2_O_2_ for 4 hours [22]. Recombinant human Irisin protein (rhIrisin) was applied at 1 μg/mL for 12 hours [23]. AICAR, an AMPK agonist, was used to mimic exercise effects at 500 μmol/L for 12 hours [24,25]. AICAR was chosen because AMPK is a central metabolic regulator activated during exercise, and pharmacological AMPK activation has been widely used as an experimental approach to reproduce the molecular effects of exercise in vitro. Thus, AICAR treatment allowed us to evaluate whether AMPK-dependent mechanisms converge with FNDC5/Irisin signaling in the regulation of oxidative stress and fibrosis. RhIrisin (R&D Systems, 067-IR), AICAR (Sigma-Aldrich, A9978), and H_2_O_2_ (Sigma-Aldrich, H1009) were purchased from the indicated suppliers. Cells were assigned to five groups: Control, H_2_O_2_, H_2_O_2_ + AICAR, H_2_O_2_ + rhIrisin, and H_2_O_2_ + rhIrisin + AICAR.

### 2.4 Echocardiographic assessment

One day after the final resistance training session, mice were anesthetized with isoflurane (mixed with oxygen at a ratio of 1:5) and placed in the supine position. Cardiac function was evaluated using a VINNO ultrasound system (VINNO, Suzhou, China). A high-frequency linear array transducer (VINNO M80, 18–22 MHz) was used, and all measurements were performed by a single experienced operator blinded to group allocation. Mice were positioned in the supine posture on a heated platform, and parasternal long-axis M-mode images were obtained at the level of the papillary muscles. Three consecutive cardiac cycles were recorded for each parameter, and the average value was used for analysis to reduce intra-operator variability. The following parameters were measured: left ventricular internal diameter at end-diastole (LVIDd), left ventricular internal diameter at end-systole (LVIDs), and ejection fraction (EF). Fractional shortening (FS) was calculated to assess cardiac function.

### 2.5 Detection of biomarkers

Blood was collected via retro-orbital puncture and centrifuged at 1,000 r/min for 5 minutes to isolate serum. Serum levels of creatinine (Scr) and blood urea nitrogen (BUN), indicators of renal injury, were quantified using commercial assay kits (Nanjing Jiancheng Bioengineering Institute). Kidney tissues were homogenized in precooled 0.86% saline (1:9, w/v), and supernatants were obtained by centrifugation for malondialdehyde (MDA) measurement using oxidative stress detection kits. All assays were conducted according to the manufacturer’s instructions, and absorbance was measured using a microplate reader (Bio-Tek Instruments).

### 2.6 Histological staining

Paraffin-embedded kidney sections were subjected to Masson’s trichrome and periodic acid–Schiff (PAS) staining and examined under an optical microscope (Olympus BX51, Japan). Collagen volume fraction (CVF) was quantified as the percentage of the tissue area stained blue. For PAS-stained sections, five random fields per section were selected to assess the integrity of the brush border of renal tubular epithelial cells, serving as an indicator of tubular damage. Tubular injury was scored based on the following criteria: 0 = normal; 1 = < 25% of tubules damaged per field; 2 = 25–50%; 3 = 50–75%; 4 = 75–100%. This semi-quantitative scoring method was adapted from established histological protocols [26], and scoring was conducted by two blinded observers. Although representative images are not provided here, detailed morphological criteria including brush border loss, nuclear dropout, and epithelial vacuolization were rigorously applied to ensure consistency. Tubular injury was defined as nuclear loss, reduced or absent brush border, epithelial swelling or vacuolar degeneration [26].

### 2.7 RT-qPCR

Total RNA was extracted from renal tissues using Trizol reagent. cDNA was synthesized using a reverse transcription kit (TaKaRa, Japan) according to the manufacturer’s protocol. qPCR amplification was performed using a SYBR Green-based PCR kit (TaKaRa). The primers (synthesized by Sangon Biotech, Shanghai, China) were as follows: FNDC5-F: 5′-GGCTGGGAGTTCATGTGGAA-3′ FNDC5-R: 5′-TGGGAAGCGGTTATCTTTGCT-3′ GAPDH-F: 5′-CAGTGCCAGCCTCGTCTCAT-3′ GAPDH-R: 5′-AGGGGCATCCACAGTCTTC-3′

### 2.8 Western blotting

Kidney tissues were minced, homogenized (Model F6/10, FLUKO), and centrifuged to collect supernatants. HKC cells were lysed post-intervention, collected by scraping, and sonicated (JY-25013, Jining Tianhua). Protein concentration was determined using a BCA kit. Samples were mixed with 5 × loading buffer and RIPA, boiled at 100°C for 10 min, and stored at –20°C until use.

Proteins were separated by SDS-PAGE, transferred to PVDF membranes, and blocked with 5% skim milk or 5% BSA for 60 min at room temperature. Primary antibodies were used at the following dilutions: FNDC5 (1:1000), TGF-β1 (1:1000), Smad2/3 (1:1000), p-Smad2/3 (1:1000), SOD1 (1:1000), SOD2 (1:1000), MMP2 (1:1000), MMP9 (1:1000), α-SMA (1:1000), Collagen-1 (1:1000), Collagen-3 (1:1000), CTGF (1:500), GAPDH (1:5000), and β-actin (1:5000). Detailed antibody information, including manufacturer and catalog numbers, is now provided as follows: FNDC5 (Abcam, ab174833), TGF-β1 (Cell Signaling Technology, #3711), Smad2/3 (Cell Signaling Technology, #8685), p-Smad2/3 (Cell Signaling Technology, #8828), SOD1 (Proteintech, 10269–1-AP), SOD2 (Abcam, ab68155), MMP2 (Abcam, ab92536), MMP9 (Abcam, ab76003), α-SMA (Sigma-Aldrich, A5228), Collagen-1 (Abcam, ab34710), Collagen-3 (Abcam, ab7778), CTGF (Santa Cruz Biotechnology, sc-14939), GAPDH (Proteintech, 60004–1-Ig), and β-actin (Sigma-Aldrich, A1978). Membranes were incubated overnight at 4°C, followed by washing and incubation with HRP-conjugated secondary antibodies for 90 min. Protein bands were visualized using the Bio-Rad ChemiDoc^TM^, MP imaging system (Universal Hood III, Bio-Rad, USA) and analyzed digitally. Densitometric analysis was performed using Image Lab 5.1 software (Bio-Rad). Arbitrary units (AU) were calculated as the ratio of target protein intensity to the corresponding GAPDH or β-actin band. For clearer visualization, all values were normalized to the mean of the control group, which was set to 1.0.

### 2.9 Data acquisition and statistical analysis

Western blot results were quantified using Image Lab 5.1 software (Bio-Rad, CA, USA). All statistical analyses and graphical outputs were performed using GraphPad Prism 8.0.2 (GraphPad Software, La Jolla, CA, USA). One-way analysis of variance (ANOVA) was used to evaluate differences among groups, followed by Tukey’s post hoc test. All data are expressed as mean ± standard error of the mean (SEM). Statistical significance was defined at P < 0.05. In addition, a priori sample size calculations were not performed; group sizes were chosen based on previous studies employing similar murine and cellular models of cardiorenal syndrome to ensure adequate statistical power. The normality of data distribution was assessed using the Shapiro–Wilk test, and homogeneity of variances was verified using Levene’s test. Outliers were identified by the Grubbs’ test (α = 0.05) and excluded only if justified by technical error. For in vitro experiments involving repeated measurements under different treatments, repeated-measures ANOVA was employed, followed by Tukey’s post hoc test for multiple comparisons. These steps were taken to minimize bias and to ensure the robustness of statistical inference.

## 3 Results

### 3.1 Resistance exercise upregulates renal FNDC5 expression and alleviates MI-induced renal injury in Type 2 CRS mice

**Fig 1:**
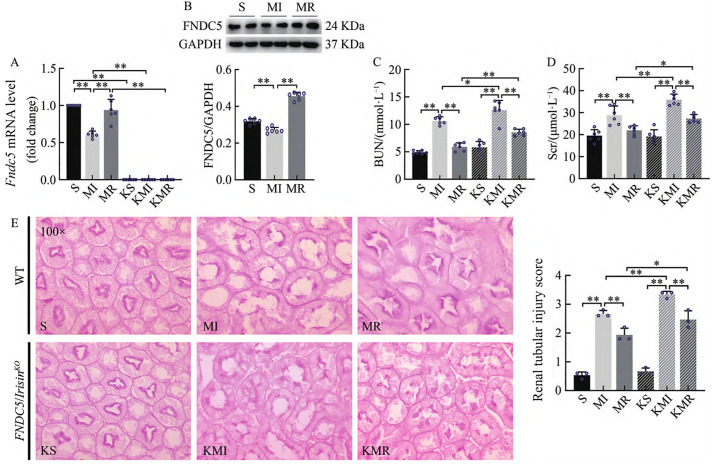
Renal FNDC5 Gene and Protein Expression, Serum BUN and Scr, And Renal PAS Staining Results in WT and *FNDC5/Irisin*^*KO*^ Mice; (A) FNDC5 mRNA levels in renal tissue; (B) Western blot analysis showing *FNDC5* and *GAPDH* protein expression. Gels were cropped from different parts of the same gel; **(C)** Serum BUN levels in different experimental groups; **(D)** Serum Scr levels in different experimental groups; **(E)** Representative histological images showing renal tubular injury (PAS staining). Renal tubular injury scores. Data are presented as mean ± SE (n = 6 per group). Statistical significance: P < 0.05, P < 0.01. Note: P < 0.05 (*), P < 0.01 (**); same below.

The expression levels of FNDC5, the precursor of Irisin, were assessed in renal tissues at both mRNA and protein levels. Compared with the sham-operated (S) group, the myocardial infarction (MI) group exhibited a significant reduction in FNDC5 gene and protein expression (P < 0.01). In contrast, mice in the myocardial infarction plus resistance exercise (MR) group showed significantly increased FNDC5 expression compared to the MI group (P < 0.01). As expected, FNDC5 gene expression was undetectable in FNDC5/Irisin knockout (KO) mice (Fig 1A–1B).

Serum creatinine (Scr) and blood urea nitrogen (BUN) are commonly used indicators of renal dysfunction and injury [27–28]. The results revealed that serum Scr and BUN levels were significantly elevated in the MI group compared to the S group (P < 0.01). Resistance exercise intervention significantly reduced these levels in Type 2 CRS mice (P < 0.01). Additionally, serum Scr and BUN levels in the knockout MI (KMI) group were significantly higher than those in the MI group (P < 0.05, P < 0.01), and levels in the knockout MR (KMR) group were significantly elevated compared to the MR group (P < 0.05, P < 0.01, Fig 1C–1D).

PAS staining was used to evaluate tubular injury in kidney tissues, with the apical brush border of renal tubules appearing red and the epithelial cells light pink. As shown in Fig 1E, kidneys from sham-operated (S) mice exhibited intact renal tubules with continuous, sharply defined brush borders. In contrast, the MI group displayed extensive tubular damage characterized by brush border attenuation or complete loss, tubular dilation, and occasional epithelial cell detachment into the lumen. These morphological alterations corresponded to significantly higher tubular injury scores in the MI group relative to the S group (P < 0.01). In the MI group, brush border loss and higher tubular injury scores were observed relative to the S group (P < 0.01). Resistance training significantly reduced tubular injury scores in the MR group compared with the MI group (P < 0.01). Notably, the KMI group displayed significantly higher tubular injury scores than the MI group (P < 0.01), and the KMR group showed elevated scores compared to the MR group (P < 0.05, Fig 1E–1F). These findings confirm the successful establishment of the Type 2 CRS model induced by MI and demonstrate that resistance exercise effectively attenuates renal tubular brush border damage and improves kidney function. Importantly, exercise capacity during the resistance training protocol was comparable between WT and KO mice, ensuring that the differences observed in renal and cardiac outcomes were not attributable to disparities in exercise compliance. Moreover, FNDC5/Irisin appears to play a key role in mediating the renoprotective effects of resistance exercise.

**Fig 2:**
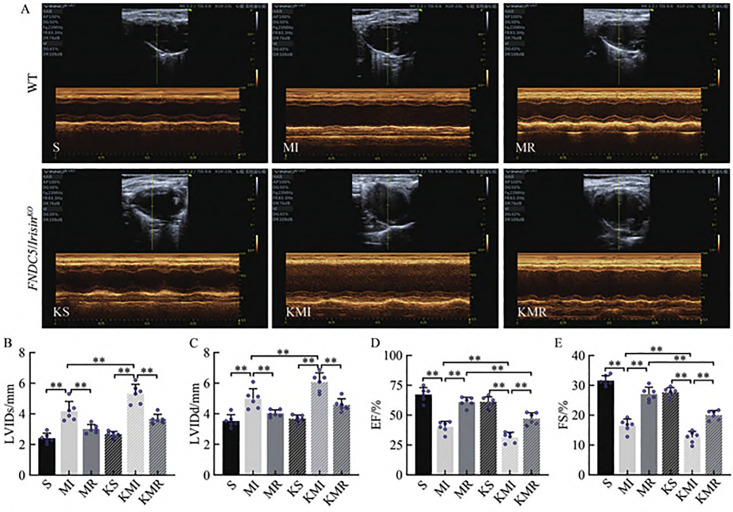
Test Results of Cardiac Function in WT and *FNDC5/Irisin*^*KO*^ Mice. **(A)** Representative echocardiographic images showing left ventricular function in different experimental groups: WT (S, MI, MR) and FNDC5/IrisinKO (KS, KMI, KMR); **(B)** Left ventricular internal diameter at end-diastole (LVIDd); **(C)** Left ventricular internal diameter at end-systole (LVIDs). (D) Ejection fraction (EF); (E) Fractional shortening (FS). Measurements were made using echocardiographic imaging to assess cardiac function.

Echocardiographic results (Fig 2) revealed that, compared with the sham-operated (S) group, mice in the myocardial infarction (MI) group exhibited significantly increased LVIDd and LVIDs (P < 0.01), along with markedly decreased ejection fraction (EF%) and fractional shortening (FS%) (P < 0.01). In contrast, the MR group showed significantly reduced LVIDd and LVIDs (P < 0.01), and significantly elevated EF% and FS% (P < 0.01), compared with the MI group. Furthermore, LVIDd and LVIDs were significantly higher in the KMI group than in the MI group (P < 0.01), while EF% and FS% were significantly lower (P < 0.01). Similarly, EF% and FS% in the KMR group were significantly lower than those in the MR group (P < 0.01). These findings confirm the successful establishment of the MI model and suggest that resistance exercise improves cardiac function in MI-induced Type 2 CRS. Moreover, FNDC5/Irisin appears to contribute to the cardioprotective effects observed during resistance training in ischemic hearts.

### 3.2 Resistance exercise suppresses renal oxidative stress and interstitial collagen deposition in Type 2 CRS mice

**Fig 3:**
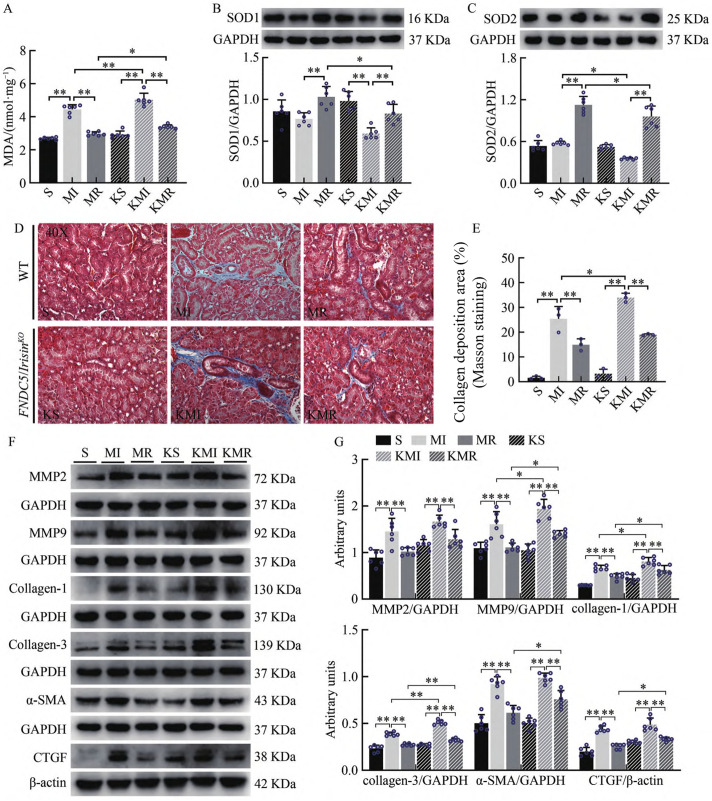
Results of Renal Oxidative Stress and Fibrosis Detection in WT and *FNDC5/Irisin*^*KO*^ Mice. **(A)** MDA levels in renal tissue; **(B)** Western blot analysis showing SOD1 protein expression. Gels were cropped from different parts of the same gel; **(C)** Western blot analysis showing SOD2 protein expression. Gels were cropped from different parts of the same gel; **(D)** Representative Masson’s trichrome staining images showing renal collagen deposition; **(E)** Quantification of collagen deposition area (%) from Masson’s staining; **(F)** Western blot analysis showing expression of MMP2, MMP9, collagen-1, collagen-3, α-SMA, and CTGF proteins. Gels were cropped from different parts of the same gel; **(G)** Quantification of protein expression levels of MMP2, MMP9, collagen-1, collagen-3, α-SMA, and CTGF. Gels were cropped from different parts of the same gel. All experiments were performed with n = 6 biological replicates per group. Data are shown as mean ± SE. P < 0.05, P < 0.01 indicate statistical significance by one-way ANOVA followed by Tukey’s post hoc test.

As shown by oxidative stress assay kits and Western blotting results, renal malondialdehyde (MDA) levels were significantly elevated in the myocardial infarction (MI) group compared with the sham-operated (S) group (P < 0.01). Resistance exercise markedly enhanced the expression of antioxidant proteins SOD1 and SOD2 and reduced MDA levels in the kidneys of Type 2 CRS mice (P < 0.01). In contrast, the knockout MI (KMI) group exhibited increased MDA content (P < 0.01) and reduced SOD2 protein expression (P < 0.05) compared with the MI group. Similarly, MDA levels were elevated (P < 0.05), and both SOD1 and SOD2 protein levels were decreased (P < 0.05) in the knockout MR (KMR) group compared with the MR group (Fig 3A–3C).

To further assess renal fibrosis, Masson’s trichrome staining and fibrosis-related protein expression were evaluated. Collagen fibers were visualized as blue deposits, indicating interstitial collagen accumulation. Representative images in Fig 3D show that renal tissue from the S group had minimal blue staining, indicating a normal interstitial collagen framework. In stark contrast, kidneys from the MI group exhibited prominent peritubular and perivascular blue staining, revealing dense, interconnected networks of collagen fibers expanding the interstitial space. Compared with the S group, the MI group showed a significant increase in renal interstitial collagen deposition and fibrosis severity (P < 0.01). Resistance exercise markedly reduced collagen accumulation in the MR group compared with the MI group (P < 0.01). Conversely, collagen deposition in the KMI group was significantly higher than in the MI group (P < 0.05, Fig 3D–3E).

Western blotting analysis revealed that, relative to the S group, the MI group exhibited significantly elevated expression of fibrosis-related proteins including MMP2, MMP9, collagen-1, collagen-3, α-SMA, and CTGF (P < 0.01). These increases were significantly attenuated following resistance exercise intervention in Type 2 CRS mice (P < 0.01). Additionally, the KMI group showed upregulated MMP9, collagen-1, and collagen-3 expression compared with the MI group (P < 0.05, P < 0.01). In the KMR group, expression levels of MMP9, collagen-1, collagen-3, α-SMA, and CTGF were significantly higher than those in the MR group (P < 0.05, P < 0.01, Fig 3F–3G). These findings indicate that resistance exercise significantly suppresses MI-induced renal interstitial collagen deposition and enhances renal antioxidant capacity in Type 2 CRS mice. Moreover, knockout of FNDC5/Irisin attenuates the inhibitory effects of resistance training on oxidative stress and fibrosis, implying that Irisin may play a role in mediating the renoprotective effects of exercise.

### 3.3 AICAR and rhIrisin Inhibit the TGF-β1–Smad2/3 Pathway to Attenuate H_2_O_2_-Induced Fibrosis in HKC Cells

**Fig 4:**
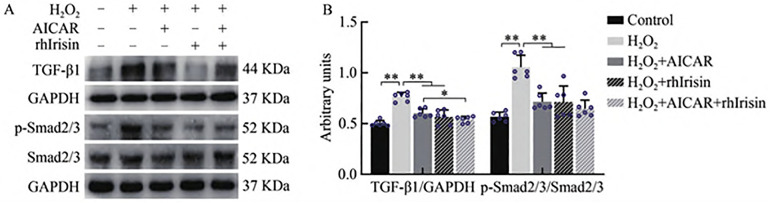
AICAR and rhIrisin Inhibited TGFβ1-Smad2/3 Signaling Pathway in HKC Cells. **(A)** Western blot analysis showing TGF-β1, p-Smad2/3, and Smad2/3 protein expression. Gels were cropped from different parts of the same gel; **(B)** Quantification of TGF-β1 and p-Smad2/3 protein expression. Gels were cropped from different parts of the same gel. Statistical analysis of in vitro data was performed using repeated-measures ANOVA with Tukey’s post hoc test.

Oxidative stress is recognized as a key trigger of fibrosis [29]. To further elucidate the mechanism by which Irisin mediates the reduction of renal interstitial collagen deposition in MI mice undergoing resistance exercise, a fibrosis model was established in HKC cells using H_2_O_2_-induced oxidative stress. The effects of Irisin and AICAR on the TGF-β1–Smad2/3 signaling pathway were subsequently examined. Western blotting results revealed that, compared with the control group, H_2_O_2_ exposure significantly increased TGF-β1 protein expression and the phosphorylation level of Smad2/3 (P < 0.01). However, treatment with AICAR and/or recombinant human Irisin (rhIrisin) significantly suppressed both TGF-β1 expression and Smad2/3 phosphorylation (P < 0.01, Fig 4). These findings indicate that in HKC cells, AICAR and/or rhIrisin are associated with reduced activation of the TGF-β1–Smad2/3 pathway induced by H2O2, which coincides with mitigation of oxidative stress–driven fibrotic responses. The inclusion of AICAR in these experiments provided a pharmacological means to mimic exercise-induced AMPK activation, allowing us to test whether the observed protective effects are attributable to AMPK–Irisin convergence rather than Irisin alone.

**Fig 5:**
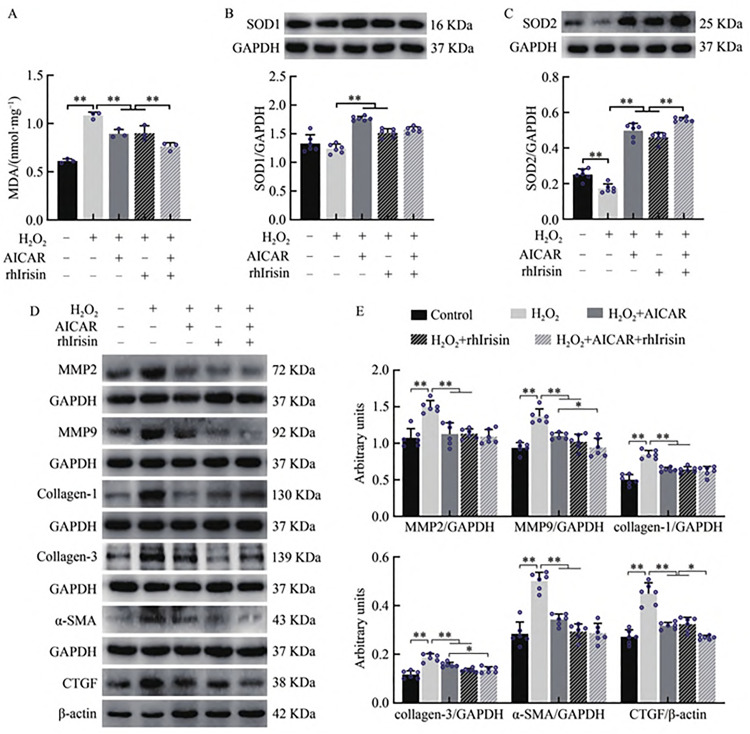
Oxidative Stress and Fibrosis in HKC Cells after AICAR and rhIrisin Intervention. **(A)** MDA levels in renal tissue; **(B)** Western blot analysis showing SOD1 protein expression. Gels were cropped from different parts of the same gel; **(C)** Western blot analysis showing SOD2 protein expression. Gels were cropped from different parts of the same gel; **(D)** Western blot analysis showing expression of MMP2, MMP9, collagen-1, collagen-3, α-SMA, and CTGF proteins. Gels were cropped from different parts of the same gel. **(E)** Quantification of protein expression levels of MMP2, MMP9, collagen-1, collagen-3, α-SMA, and CTGF. Gels were cropped from different parts of the same gel. Minor variation in β-actin intensity was noted; blots shown are representative of at least three independent experiments with consistent trends. Statistical analysis of in vitro data was performed using repeated-measures ANOVA with Tukey’s post hoc test.

To evaluate the effects of Irisin and AICAR on the antioxidant capacity and collagen synthesis in HKC cells, oxidative stress assay kits and Western blotting were used to measure the levels of MDA, SOD1, SOD2, MMP2, MMP9, collagen-1, collagen-3, α-SMA, and CTGF following H_2_O_2_ exposure. The results demonstrated that, compared with the control group, H_2_O_2_ intervention significantly decreased SOD2 protein expression (P < 0.01) while markedly increasing MDA levels and the protein expression of MMP2, MMP9, collagen-1, collagen-3, α-SMA, and CTGF (P < 0.01). Intervention with AICAR or rhIrisin after oxidative stress significantly enhanced the expression of antioxidant enzymes SOD1 and SOD2 (P < 0.01), and concurrently reduced MDA content and the expression of fibrosis-related proteins (P < 0.01). Furthermore, compared with the H_2_O_2_ + AICAR group, the H_2_O_2_ + rhIrisin + AICAR group exhibited increased SOD2 expression (P < 0.01) and significantly decreased levels of MDA, MMP9, collagen-3, and CTGF (P < 0.05, P < 0.01). Similarly, relative to the H_2_O_2_ + rhIrisin group, the combined treatment group (H_2_O_2_ + rhIrisin + AICAR) showed enhanced SOD2 expression (P < 0.01) and reduced MDA and CTGF levels (P < 0.05, P < 0.01) (Fig 5). These findings suggest that AICAR and/or rhIrisin effectively attenuate oxidative stress and suppress the expression of fibrosis-associated proteins in HKC cells under oxidative injury conditions.

### 3.4 Resistance Exercise inhibits activation of the renal TGF-β1–Smad2/3 signaling pathway in Type 2 CRS mice

**Fig 6:**
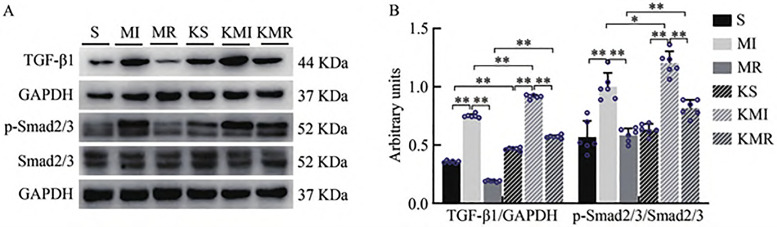
Renal TGFβ1-Smad2/3 Pathway in WT and *FNDC5/Irisin*^*KO*^ Mice. **(A)** Western blot analysis showing TGF-β1, p-Smad2/3, and Smad2/3 protein expression. Gels were cropped from different parts of the same gel; **(B)** Quantification of TGF-β1 and p-Smad2/3 protein expression. Gels were cropped from different parts of the same gel.

Western blotting results revealed that, compared with the sham-operated (S) group, the myocardial infarction (MI) group exhibited significantly elevated expression of TGF-β1 and phosphorylation of Smad2/3 in kidney tissues (P < 0.01). These increases were markedly attenuated in the myocardial infarction plus resistance exercise (MR) group (P < 0.01). In addition, the knockout sham (KS) group showed significantly higher TGF-β1 expression than the S group (P < 0.01). The knockout MI (KMI) group displayed significantly increased levels of TGF-β1 and phosphorylated Smad2/3 compared with the MI group (P < 0.05, P < 0.01). Similarly, both TGF-β1 expression and Smad2/3 phosphorylation were significantly higher in the knockout MR (KMR) group than in the MR group (P < 0.01, Fig 6). These findings suggest that resistance exercise suppresses MI-induced activation of the renal TGF-β1–Smad2/3 signaling pathway in Type 2 CRS. However, the inhibitory effect of resistance exercise on this profibrotic pathway is significantly weakened in the absence of FNDC5/Irisin, highlighting its regulatory role in exercise-mediated renal protection.

### 3.5 Schematic summary of the proposed mechanism

**Fig 7:**
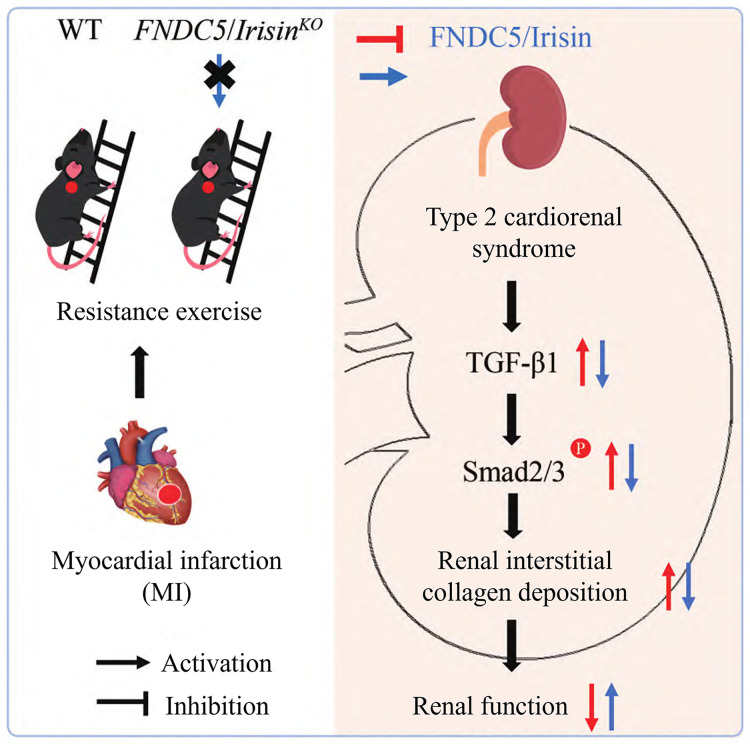
Schematic illustration of the proposed mechanism. Myocardial infarction (MI) induces renal oxidative stress and activates the profibrotic TGF-β1/Smad2/3 signaling pathway, leading to interstitial collagen deposition and renal dysfunction. Resistance exercise upregulates renal FNDC5/Irisin expression. Irisin, potentially in concert with exercise-induced AMPK activation, attenuates oxidative stress and inhibits TGF-β1/Smad2/3 pathway activation, thereby reducing renal fibrosis and improving function. Solid lines indicate pathways supported by direct evidence from this study; dashed lines indicate proposed or associative links.

Based on the in vivo and in vitro findings presented above, we propose a working model summarizing the potential mechanism by which resistance exercise confers renoprotection in Type 2 CRS (Fig 7).

## 4. Discussion

Prolonged myocardial infarction (MI) can cause damage to distant organs, including the kidneys. Persistent renal ischemia and hypoxia promote oxidative stress and inflammatory responses, leading to the activation and migration of myofibroblasts and excessive extracellular matrix (ECM) accumulation. These events contribute to renal interstitial collagen deposition and apoptosis, ultimately resulting in renal interstitial fibrosis (RIF) and functional deterioration [10,30,31]. Thus, mitigating MI-induced remote organ injury, particularly renal dysfunction, is critical for improving the quality of life in patients with heart failure. Exercise has been shown to reduce the risk of progression from heart failure to chronic kidney disease (CKD) [32], improve renal function in CKD patients [11,33], enhance physical capacity, and improve overall well-being [34–35].

### 4.1 Resistance exercise upregulates renal FNDC5/Irisin, attenuates oxidative stress and collagen deposition, and improves renal function in Type 2 CRS mice

Previous studies have reported that chronic kidney disease (CKD) leads to tubular epithelial cell swelling, tubular dilation, and brush border loss, exacerbating renal injury [36] and elevating serum BUN and Scr levels [37]. Resistance exercise has been demonstrated to alleviate tubular injury and reduce serum BUN and Scr levels, thereby improving renal function in CKD models [38]. Although aerobic exercise has been reported to ameliorate renal injury in Type 2 CRS mice [12], the effects of resistance exercise on this condition had not been previously documented. Our results demonstrated that resistance exercise significantly reduced serum BUN and Scr levels and improved tubular morphology and renal function, indicating a renoprotective effect in Type 2 CRS.

Irisin, a secreted exercise-induced myokine, exerts biological effects through autocrine, paracrine, and endocrine mechanisms [16,18]. FNDC5, the precursor of Irisin, is expressed in multiple tissues, including skeletal muscle, heart, brain, kidney, liver, and adipose tissue [39]. Clinical studies have reported decreased circulating Irisin levels in patients with cardiovascular or renal diseases [40–41]. Consistent with prior findings [12], our study confirmed that resistance training upregulated FNDC5 gene and protein expression in the kidneys of Type 2 CRS mice. Recombinant Irisin administration has been shown to reduce serum BUN and Scr levels and ameliorate renal injury in ischemia-reperfusion models [42]. In our study, FNDC5/Irisin knockout mice exhibited increased serum BUN and Scr levels, more severe renal damage, and attenuated exercise-induced renoprotection. Irisin has been shown to act directly on renal tubular epithelial cells [23,42], suggesting that exercise-induced Irisin may contribute to renal protection via multiple regulatory modes. Recent evidence indicated that Irisin confers organ protection via activation of the AMPK-SIRT1 pathway and attenuation of mitochondrial oxidative stress, thereby limiting renal and cardiovascular injury [44]. This mechanistic insight supported present findings that resistance exercise, through FNDC5/Irisin upregulation, alleviated oxidative stress and fibrosis in the kidney.

Oxidative stress plays a central role in renal structural damage, functional decline, and progression of RIF [43–45]. Studies have shown that reduced renal blood flow during heart failure leads to hypoxia and an imbalance in oxidative/antioxidative systems, promoting oxidative stress and accelerating renal dysfunction. Irisin administration in acute kidney injury models enhances antioxidant enzyme activities, such as superoxide dismutase (SOD), and alleviates oxidative damage [38]. Resistance training has been shown to increase circulating Irisin levels and elevate renal antioxidant enzyme activity in mice with kidney injury. In our study, MI induced significant renal oxidative stress, evidenced by elevated MDA levels and reduced SOD1 expression. Irisin deficiency diminished the antioxidative effects of resistance exercise, indicating that Irisin mediates resistance exercise–induced enhancement of SOD1 and SOD2 expression and reduction of MDA, thereby improving renal antioxidant capacity in Type 2 CRS mice.

RIF is a hallmark pathological feature of Type 2 CRS and a major driver of renal dysfunction [36]. Hypoxia and mechanical stress stimulate the expression of connective tissue growth factor (CTGF), promoting ECM accumulation, increased synthesis of collagen types I and III, and degradation of the tubular basement membrane by MMP2 and MMP9. These changes induce epithelial–mesenchymal transition (EMT) and interstitial collagen deposition, thereby accelerating RIF. Studies have shown increased CTGF and α-SMA expression and ECM accumulation in the kidneys of rats with chronic heart failure, while 10 weeks of resistance exercise reduces renal fibrosis in CKD rats [13]. Consistently, our study revealed that MI elevated renal expression of collagen-1/3, MMP2/9, CTGF, and α-SMA, which were significantly suppressed by resistance exercise. Recombinant Irisin has also been reported to inhibit Ang II–induced expression of α-SMA, collagen-1, and collagen-3 in the heart, alleviating myocardial fibrosis [19]. Our data showed that deletion of FNDC5/Irisin increased fibrosis marker expression and attenuated the antifibrotic effects of resistance exercise, indicating that FNDC5/Irisin plays a crucial role in mitigating renal collagen deposition in Type 2 CRS [26]

### 4.2 Potential mechanism: Resistance exercise attenuates interstitial collagen deposition via FNDC5/Irisin-mediated inhibition of the TGF-β1–Smad2/3 pathway

Overactivation of the TGF-β1–Smad signaling pathway is a key contributor to renal interstitial collagen deposition and RIF. Upon binding to TGF-β receptor II (TGFβRII), TGF-β1 activates TGFβRI kinase, which phosphorylates downstream Smad2/3. These phosphorylated Smads form complexes with Smad4 and translocate into the nucleus to regulate target gene transcription, promoting fibrosis.

Immunohistochemistry has shown that Irisin is predominantly expressed in renal tubules. H_2_O_2_-induced oxidative stress promotes EMT in tubular epithelial cells, with increased expression of EMT markers including type I collagen, vimentin, and N-cadherin [22]. In our HKC cell fibrosis model, H_2_O_2_ treatment significantly decreased SOD2 levels while increasing MDA, collagen-1/3, MMP2/9, α-SMA, and CTGF, indicating the induction of oxidative stress and fibrosis. Although NAC treatment was not included, the H2O2-based model and the redox-modulating effects of rhIrisin and AICAR collectively support the notion that oxidative stress is a primary driver of the observed fibrotic response.

Exercise has been shown to suppress TGF-β1–Smad2/3 pathway activation and renal fibrosis in hypertensive rats. In our study, both AICAR and rhIrisin significantly reduced oxidative stress markers and fibrosis-related proteins in H_2_O_2_-treated HKC cells and inhibited TGF-β1–Smad2/3 pathway activation. In vivo, knockout of FNDC5/Irisin impaired the ability of resistance exercise to suppress this signaling pathway. Prior evidence suggests that Irisin may competitively bind to TGFβRII, antagonizing TGF-β1 signaling and thus mitigating fibrosis [23]. Therefore, we hypothesize that Irisin may attenuate Smad2/3 phosphorylation, potentially through interference with TGFβRII binding, contributing to resistance exercise–mediated suppression of the TGF-β1–Smad2/3 pathway in Type 2 CRS, as summarized in our proposed model (Fig 7). It should be noted, however, that resistance exercise still exerted partial protective effects in FNDC5/Irisin KO mice compared with MI controls, indicating that FNDC5/Irisin is not the only mediator of exercise-induced renoprotection. Rather, FNDC5/Irisin appears to act as an important amplifier of these benefits, while other pathways such as AMPK activation may also contribute. This interpretation is further supported by the observation that AICAR treatment mimicked the effects of Irisin in vitro. Thus, the absence of FNDC5 primarily exacerbates myocardial infarction outcomes, whereas its presence enhances the renoprotective and antifibrotic actions of resistance training. It is also worth noting that other signaling pathways, such as MAPK, NF-κB, and PI3K/AKT, have been implicated in renal oxidative stress and fibrosis. Although not explored in the present study, potential crosstalk between these pathways and TGF-β1-Smad signaling may contribute to the phenotype observed and warrants further investigation.

Clinically, resistance exercise has been increasingly applied in patient rehabilitation programs [25]. However, it may not be suitable for individuals with severe conditions such as uncontrolled hypertension or ventricular arrhythmias. Therefore, patients should undergo clinical evaluation before initiating exercise training, and a personalized exercise prescription—taking into account frequency, intensity, duration, exercise type, and individual preferences—should be developed accordingly. Findings from the present study indicate that appropriate resistance exercise upregulates FNDC5/Irisin expression, thereby improving both cardiac and renal function in MI mice. It is thus speculated that resistance training may serve as a potential rehabilitation strategy for heart failure patients with concurrent renal injury, and that FNDC5/Irisin may represent a key molecular target mediating this therapeutic benefit.

### 4.3 Study limitations and future perspectives

While the global FNDC5/Irisin knockout mouse model provided valuable insights into the role of this myokine in exercise-induced renoprotection, several limitations should be acknowledged. First, as a systemic knockout, the model does not allow us to distinguish between the direct effects of Irisin on renal tubular epithelial cells and its indirect effects mediated through improved cardiac function, altered skeletal muscle metabolism, or modulated systemic inflammation. For instance, the attenuated cardioprotection observed in KO mice (Fig 2) could secondarily influence renal perfusion and stress, confounding the interpretation of a kidney-specific mechanism.

Second, Irisin is known to exert pleiotropic effects on multiple organs, including the heart, liver, and adipose tissue. Therefore, the phenotypic differences observed in KO mice may result from a combination of renal and extra-renal alterations. Although our in vitro experiments using HKC cells support a direct, renal tubular action of Irisin, they cannot fully recapitulate the complex in vivo milieu.

Third, an important alternative explanation for our findings must be considered. As demonstrated in Fig 2, resistance exercise significantly improved cardiac function (e.g., ejection fraction) in MI mice. Therefore, the observed renoprotection—including reduced fibrosis and oxidative stress—may be partially attributable to improved systemic hemodynamics and renal perfusion secondary to enhanced cardiac output, rather than solely to a direct renal action of FNDC5/Irisin. This hemodynamic improvement represents a plausible and significant contributor to the overall benefit.

However, several lines of evidence suggest that FNDC5/Irisin also plays a direct or amplifying role: (i) the in vitro protective effects of rhIrisin on HKC cells under oxidative stress occur independently of cardiac function or hemodynamics; (ii) within the MR group (which experienced cardiac improvement), renal outcomes were still significantly worse in Irisin-deficient (KMR) mice compared to WT (MR) mice, implying an Irisin-dependent component beyond cardiac function; and (iii) Irisin deficiency exacerbated renal injury (KMI vs. MI) even under comparable levels of post-MI cardiac dysfunction. Thus, we propose that the renoprotection observed in this model likely results from a combination of exercise-induced improvements in central hemodynamics and direct/indirect tissue-protective actions mediated by FNDC5/Irisin. Future studies measuring renal blood flow and glomerular filtration rate directly, alongside targeted manipulations, would be valuable to disentangle the relative contributions of these mechanisms.

Future studies employing kidney-specific conditional knockout or knockdown models would be instrumental in dissecting the cell-autonomous actions of renal FNDC5/Irisin. Additionally, measuring circulating and tissue-specific levels of inflammatory cytokines, metabolic markers, and other exercise-responsive myokines in both WT and KO mice could help delineate the systemic versus local contributions of the FNDC5/Irisin axis. Despite these limitations, our combined in vivo and in vitro approach provides compelling associative evidence that FNDC5/Irisin is an important amplifier of the renoprotective benefits conferred by resistance training in Type 2 CRS.

## 5. Conclusion

FNDC5/Irisin plays a critical role in suppressing renal oxidative stress and interstitial collagen deposition. Resistance exercise promotes the endogenous expression of FNDC5/Irisin in the kidney, which is correlated with reduced oxidative stress, enhanced antioxidant capacity, and attenuated activation of the TGF-β1–Smad2/3 signaling pathway. These effects collectively contribute to the attenuation of myocardial infarction–induced renal interstitial collagen deposition and the improvement of renal function.

## Supporting information

S1 File Raw image. (PDF)
