# Supplementary material for: FNDC5/Irisin-dependent renoprotection of resistance training in myocardial infarction–induced Type 2 cardiorenal syndrome
Source: PLoS One. 2026 Feb 20;21(2):e0342468. doi: 10.1371/journal.pone.0342468 (PMC12923059; doi:10.1371/journal.pone.0342468)

Figure1 FNDC5

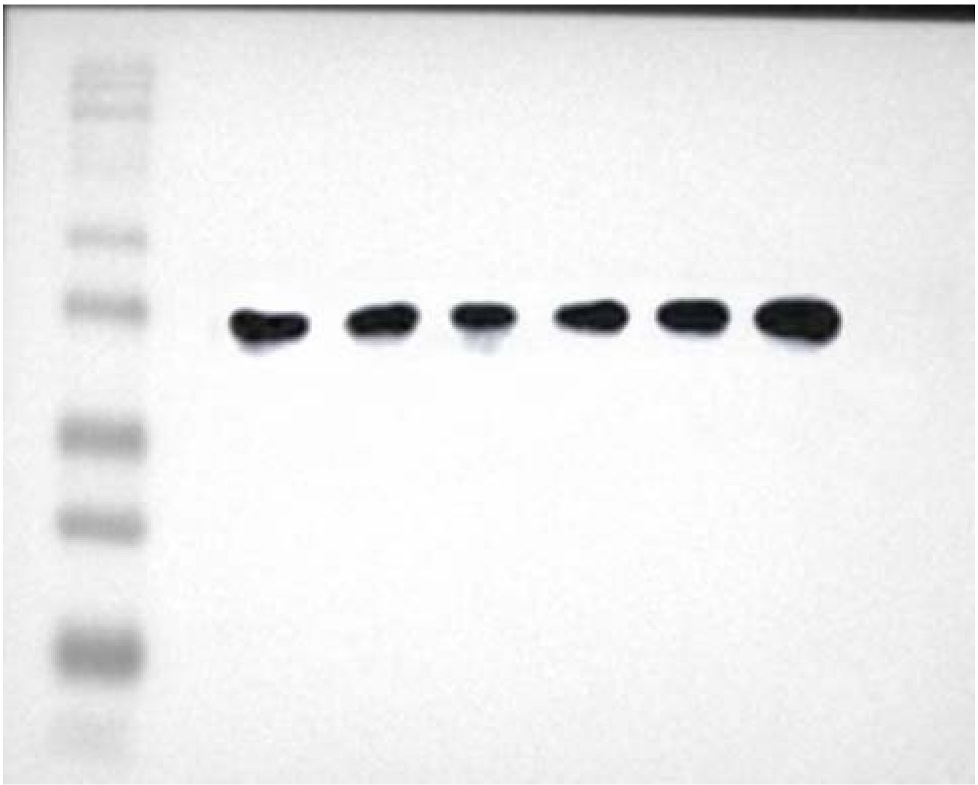

Figure1 GAPDH

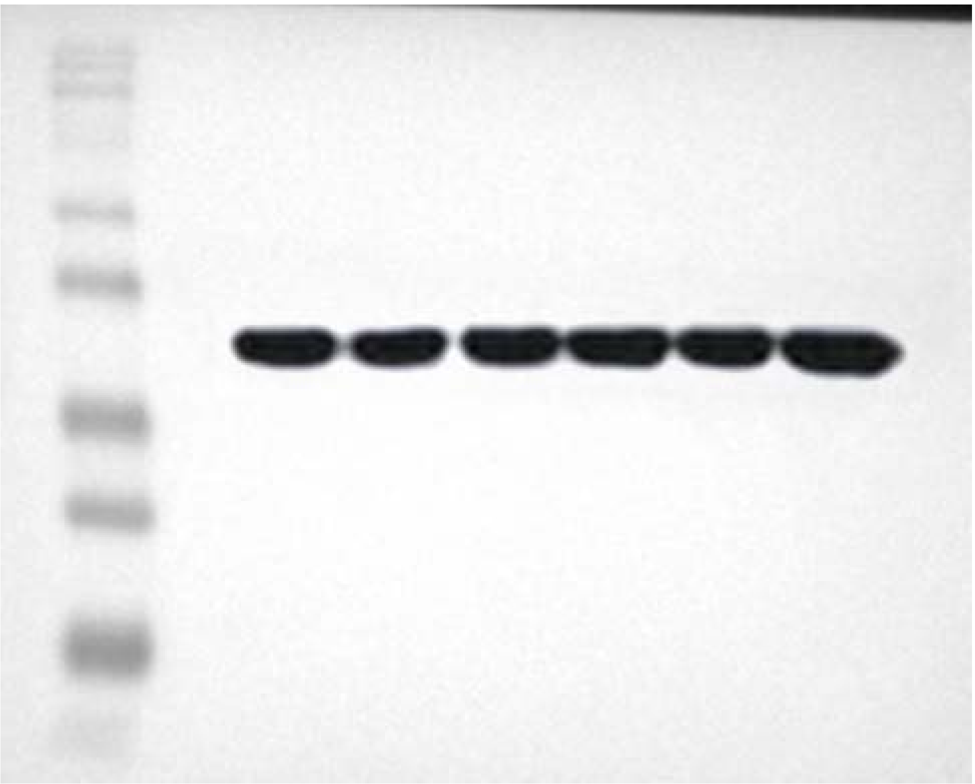

Figure3 F3 GAPDH

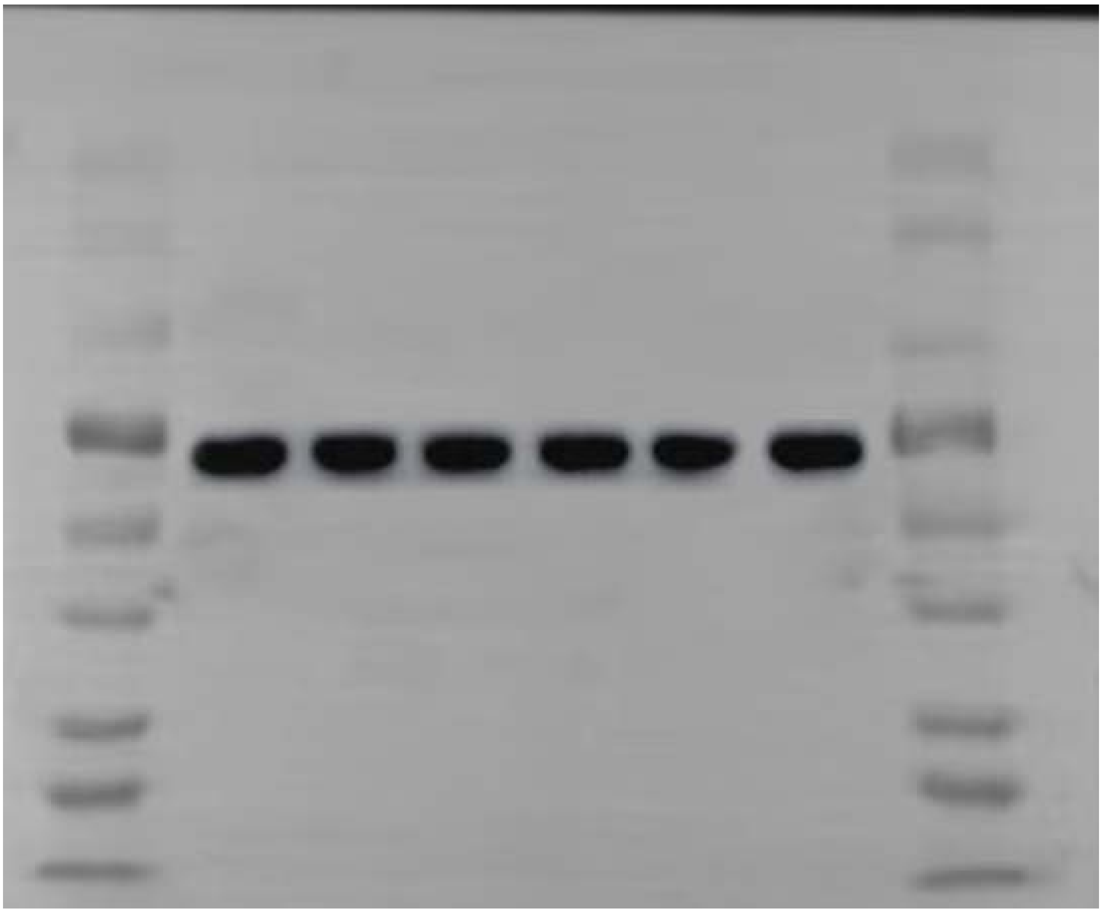

Figure3 F4 GAPDH

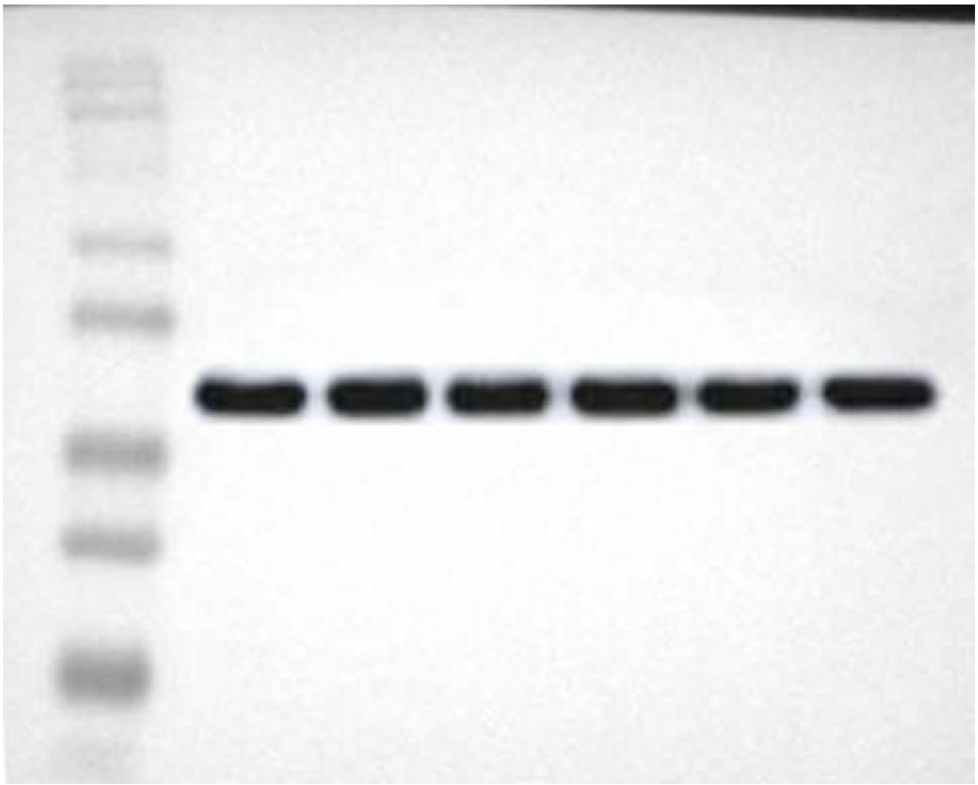

Figure3 MMP2

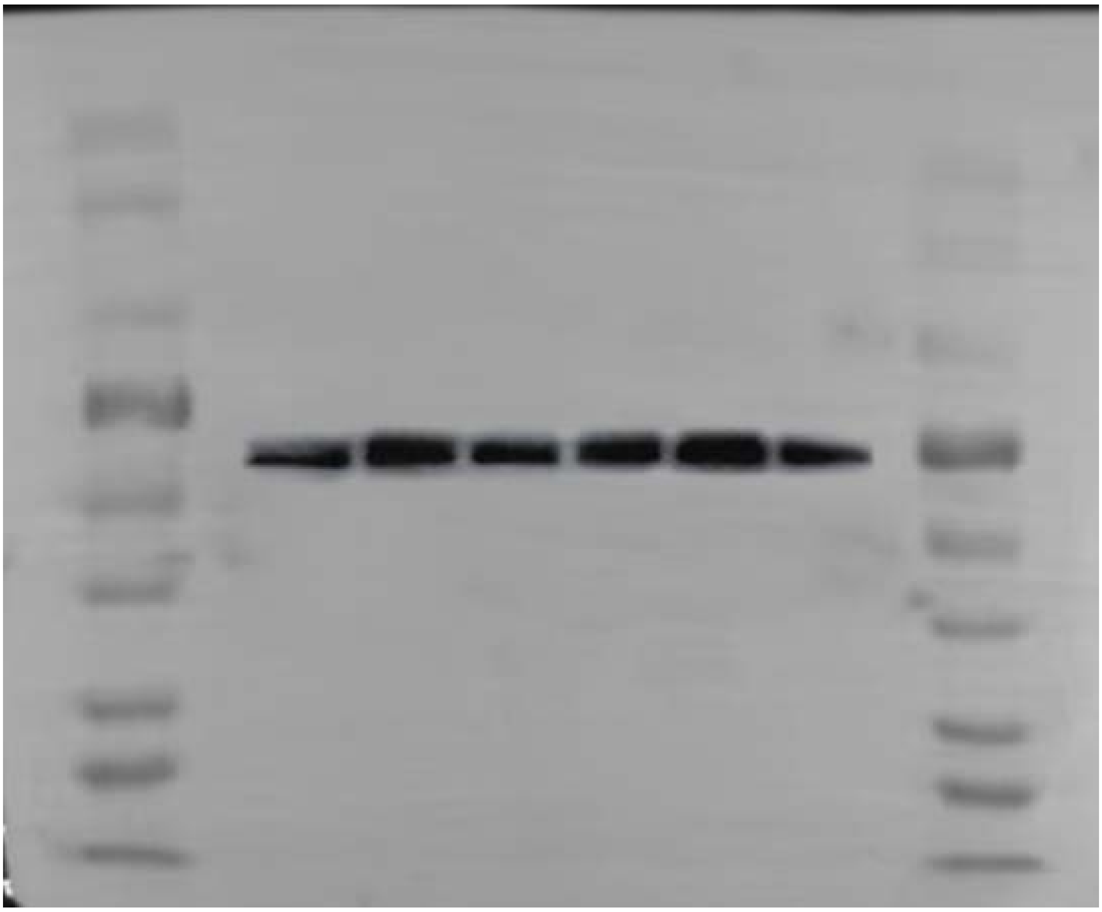

Figure3 SOD1

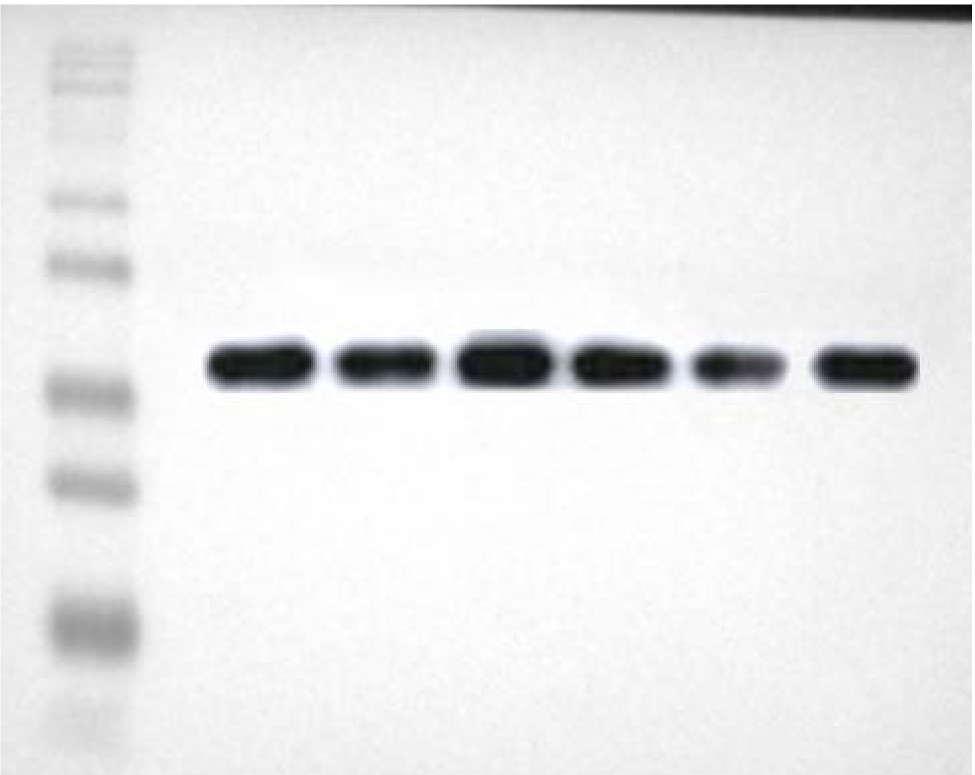

Figure3 SOD2

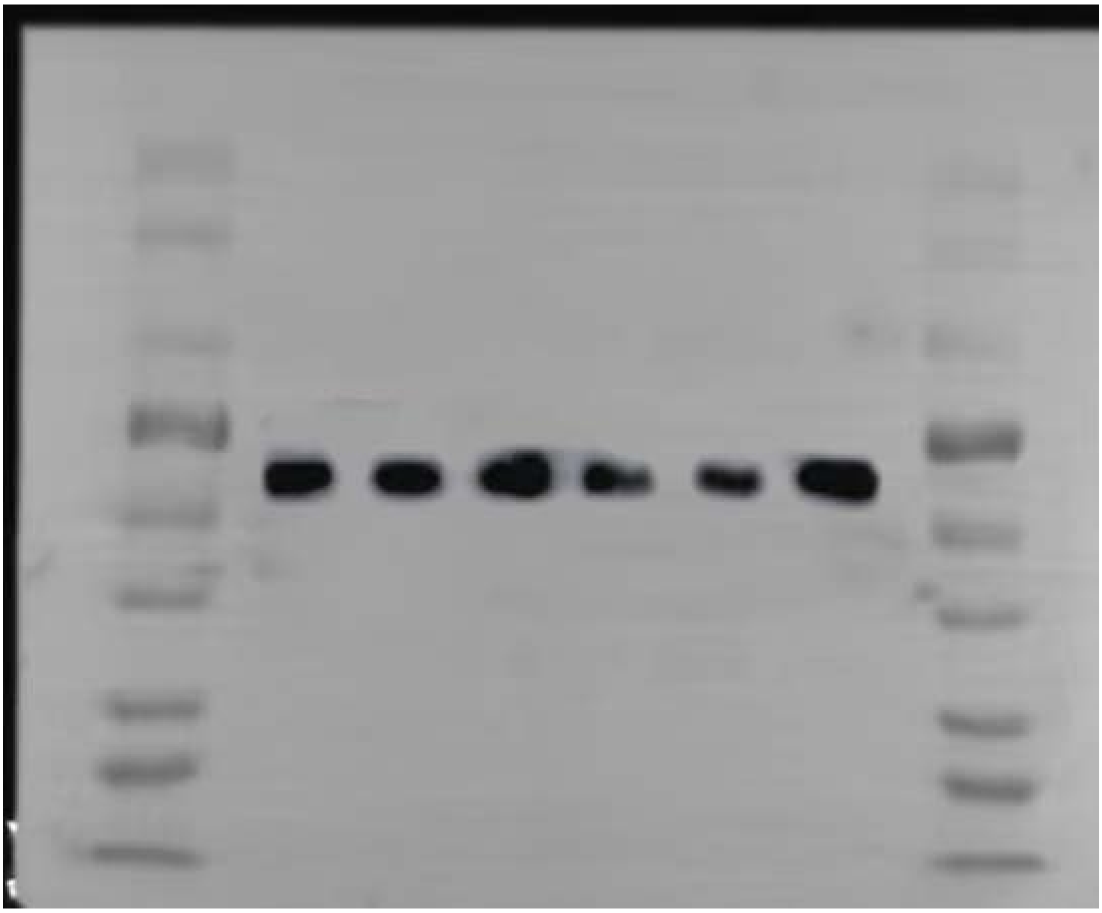

Figure3B GAPDH

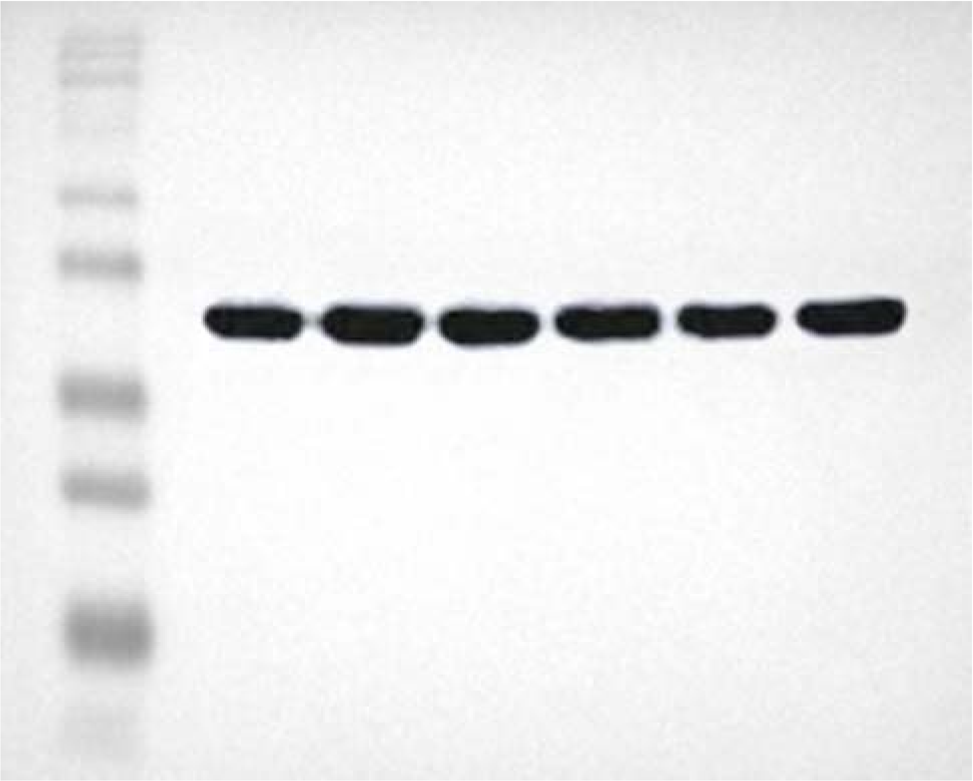

Figure3C GAPDH

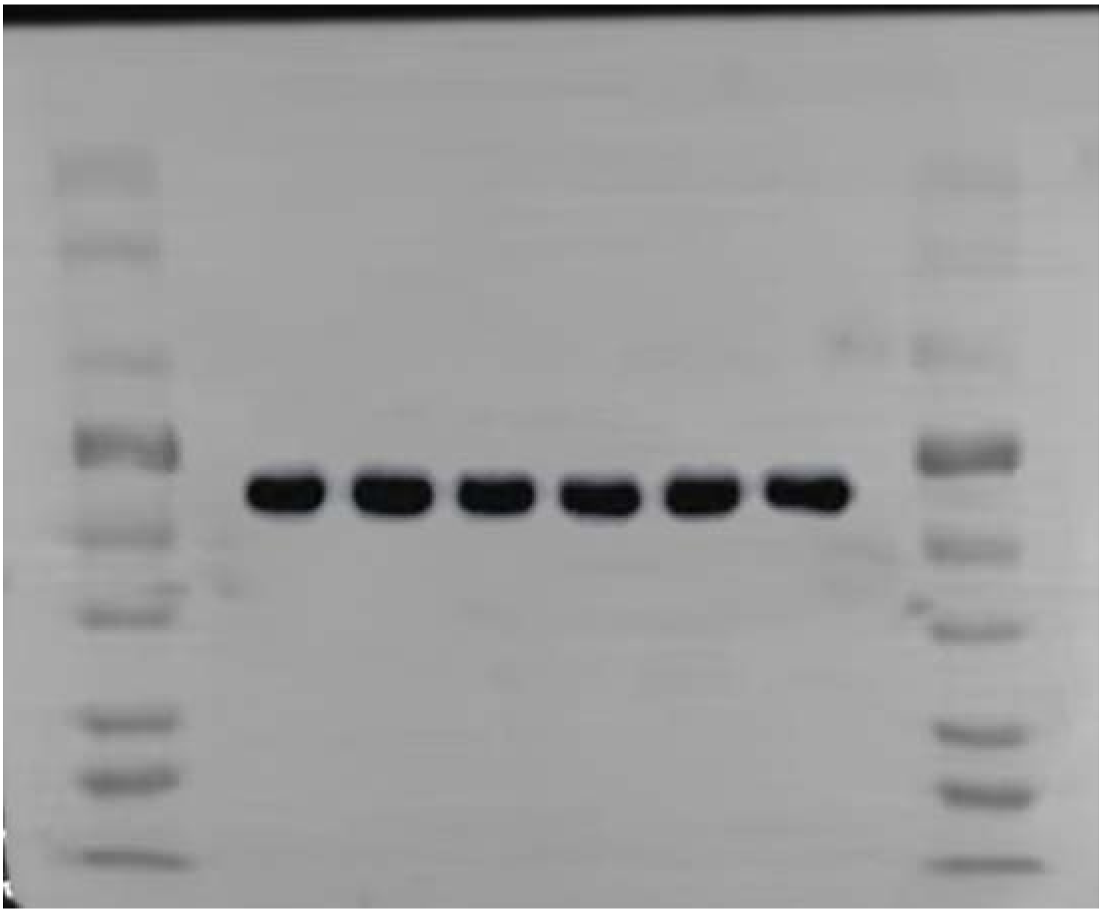

Figure3F collagren-3

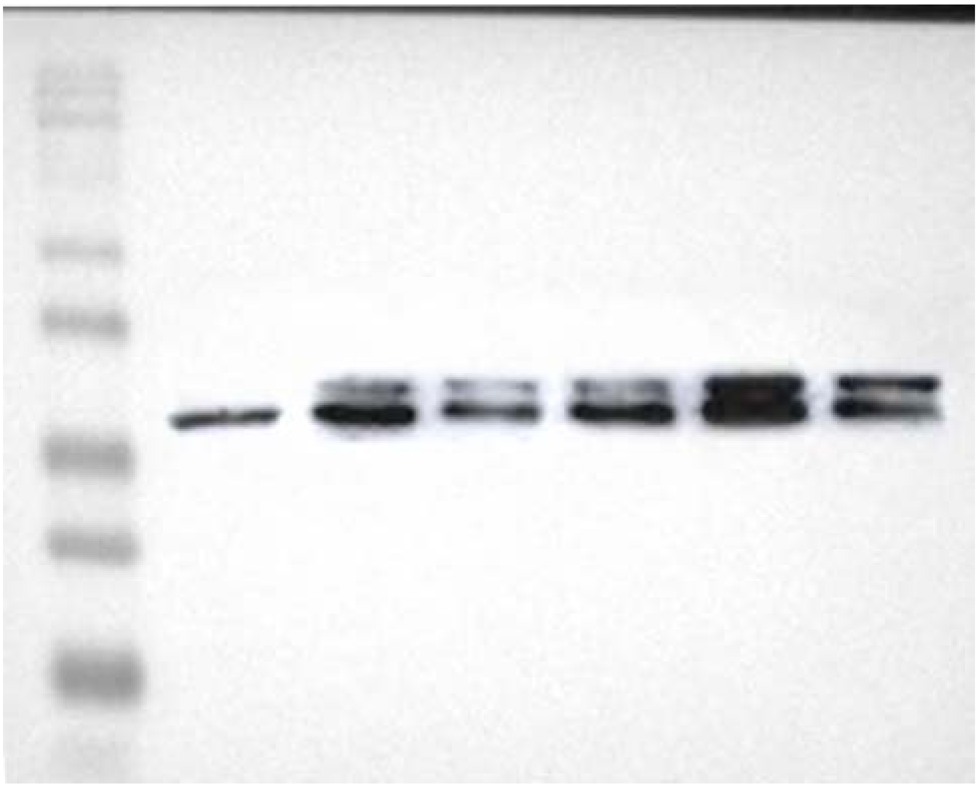

Figure3F CTGF

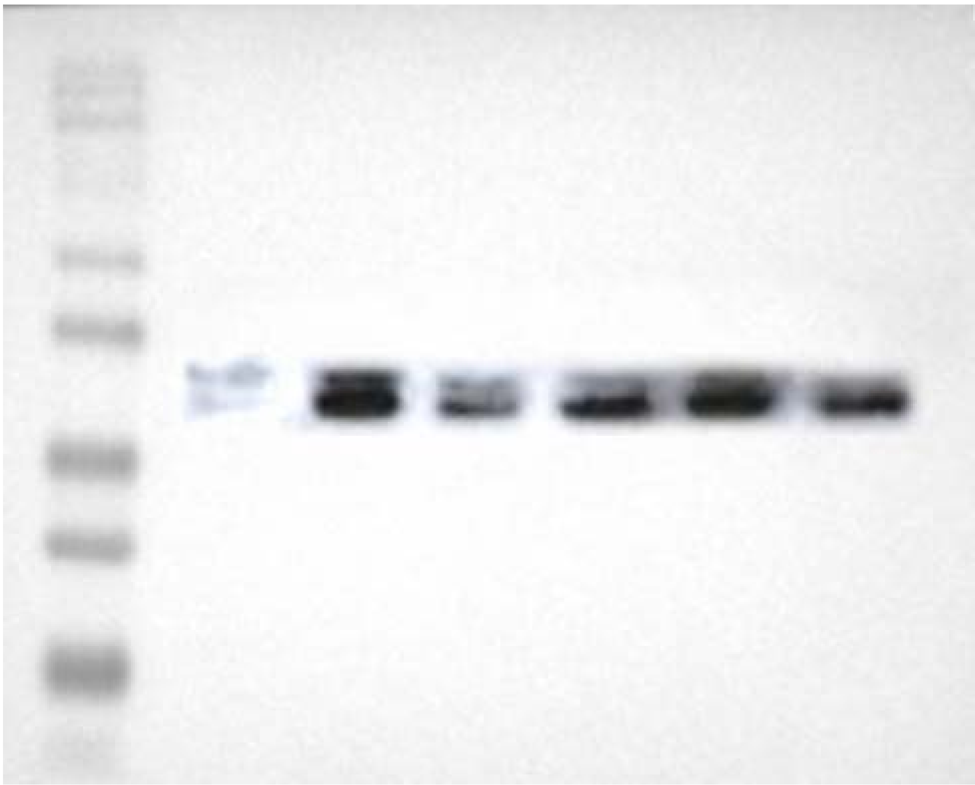

Figure3F MMP9

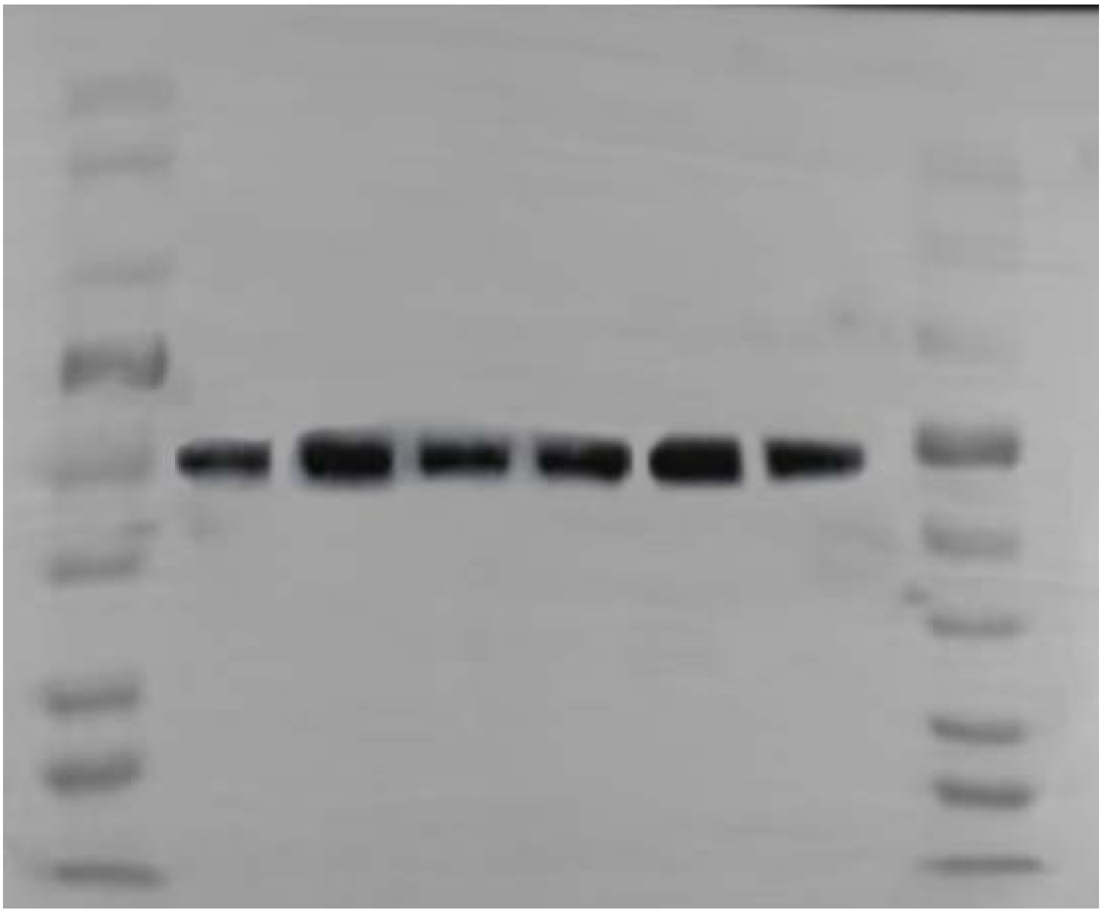

Figure3F α-SMA

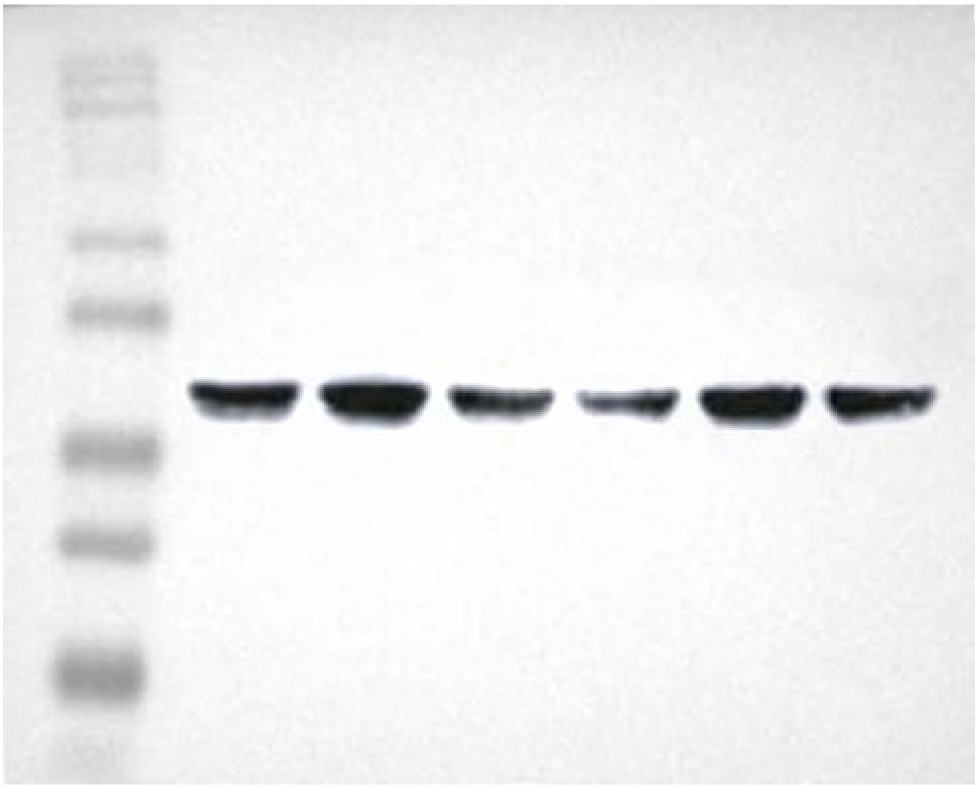

Figure3F β-actin

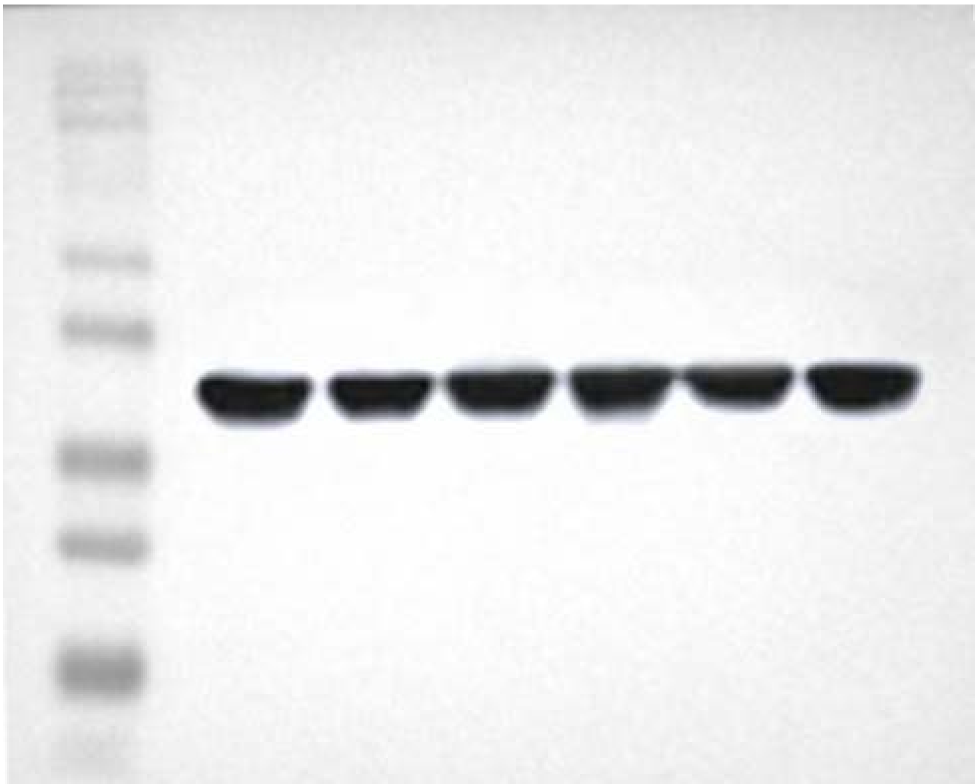

Figure3F5 GAPDH

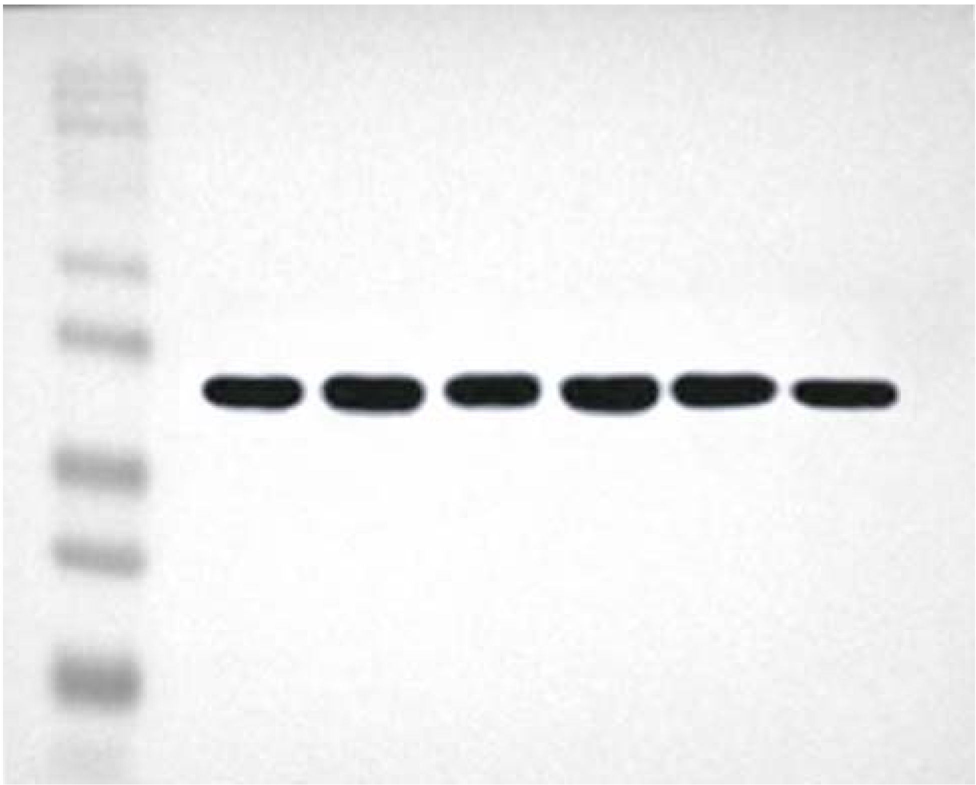

Figure4A GAPDH

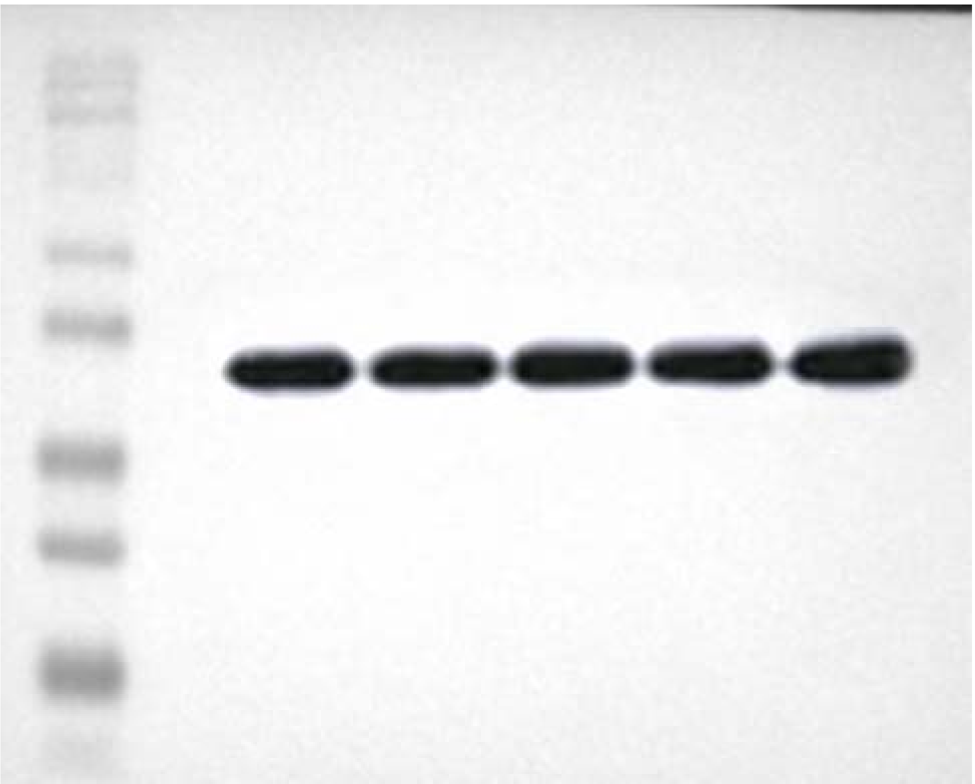

Figure4A p-Smad23

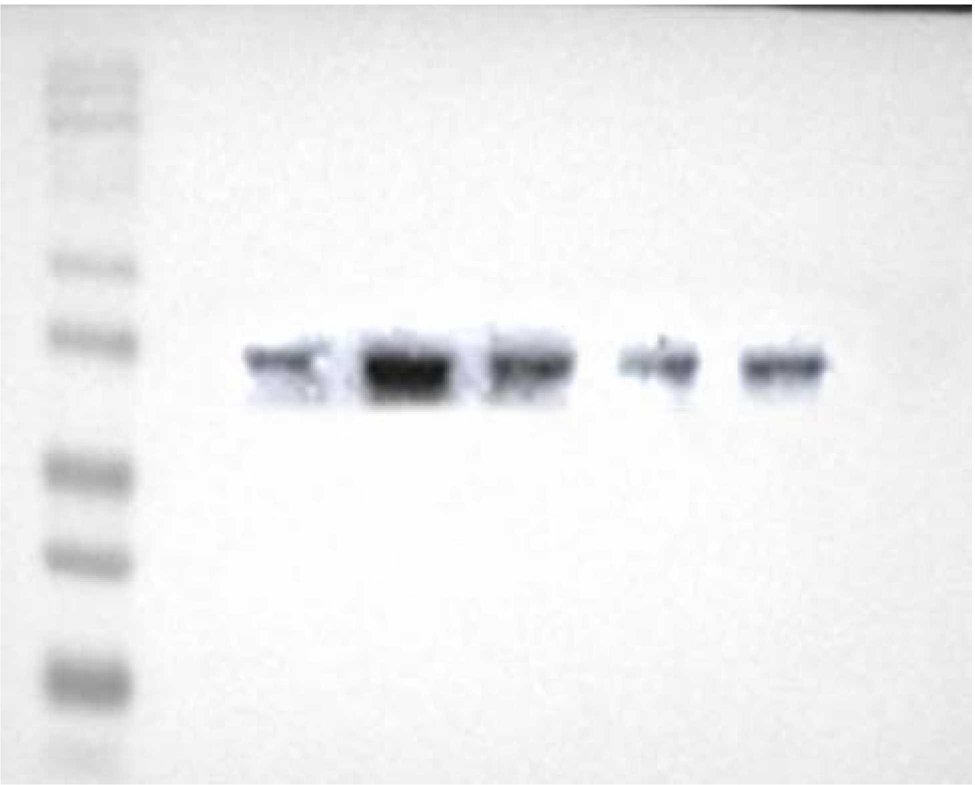

Figure4A Smad23

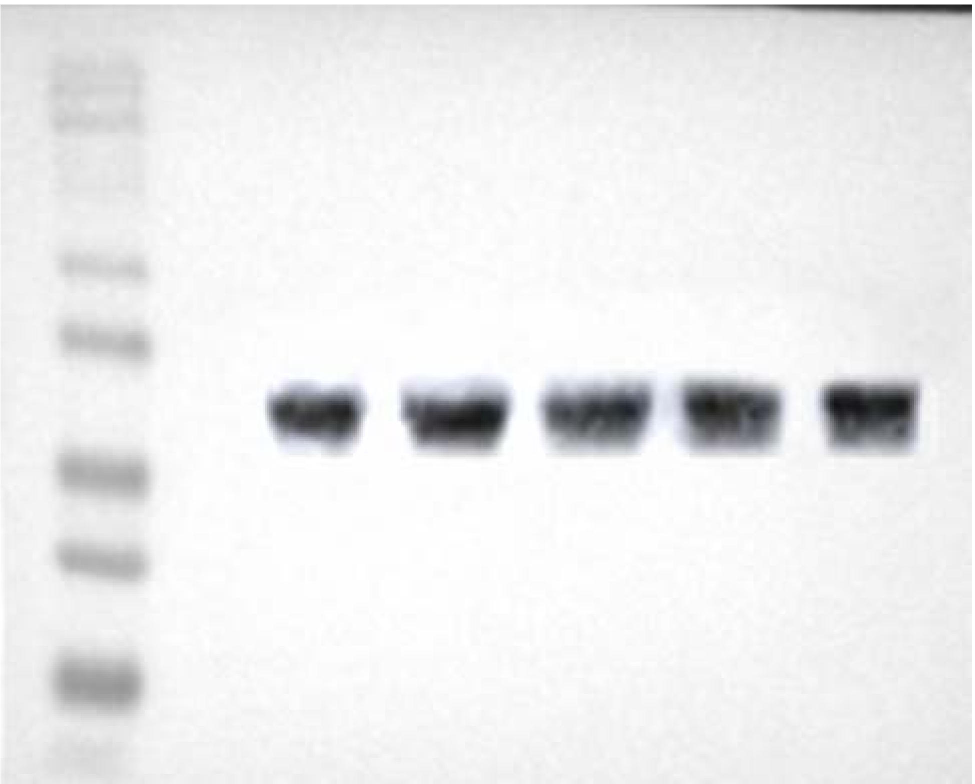

Figure4A TGF-β1

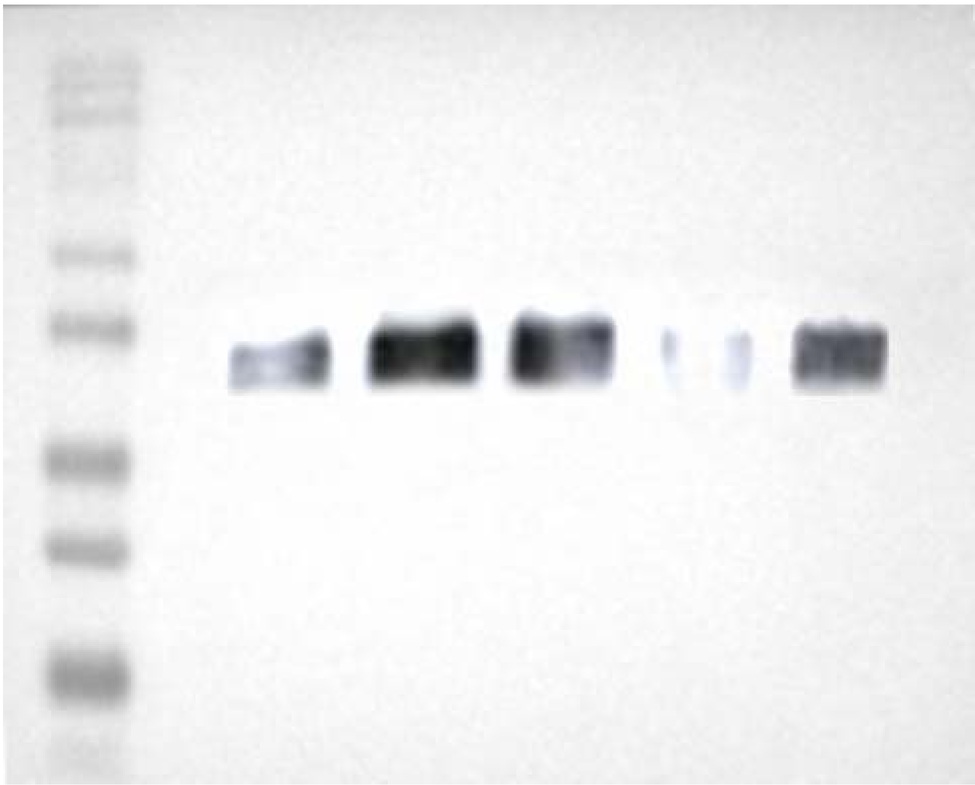

Figure4A2 GAPDH

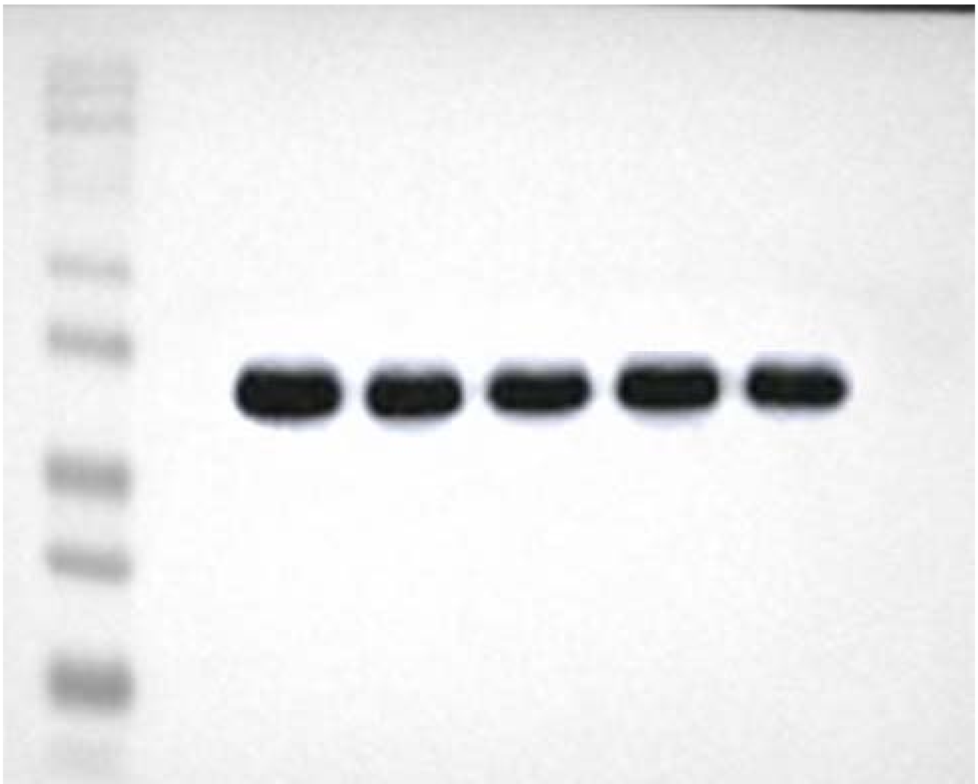

Figure5B GAPDH

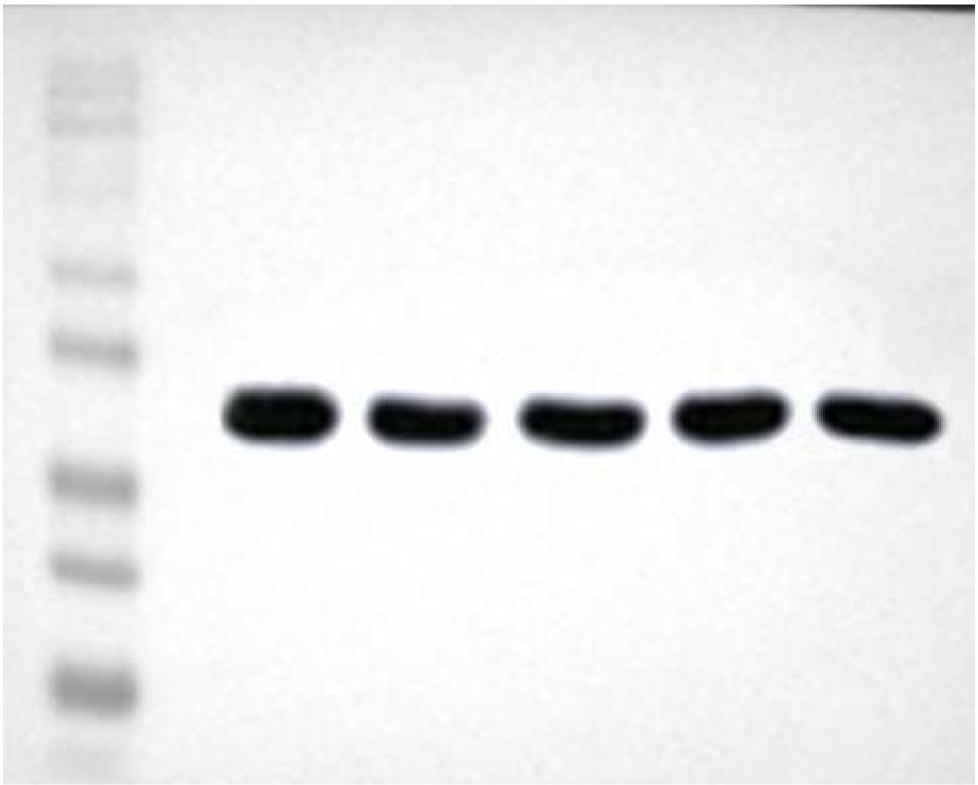

Figure5B SOD1

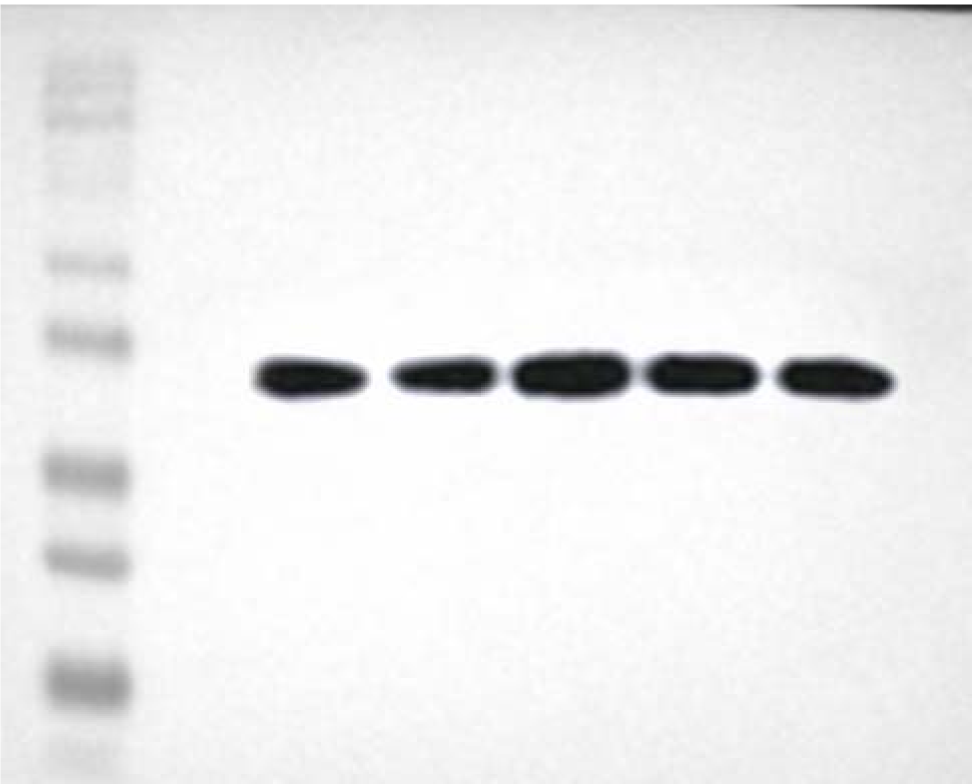

Figure5C GAPDH

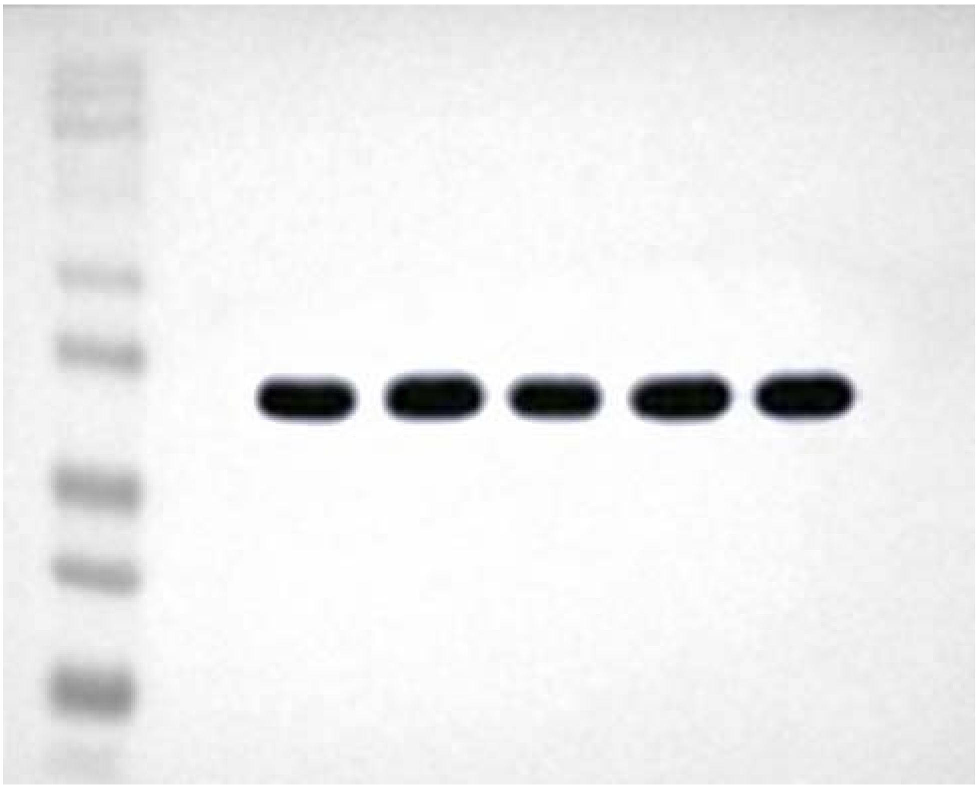

Figure5C SOD2

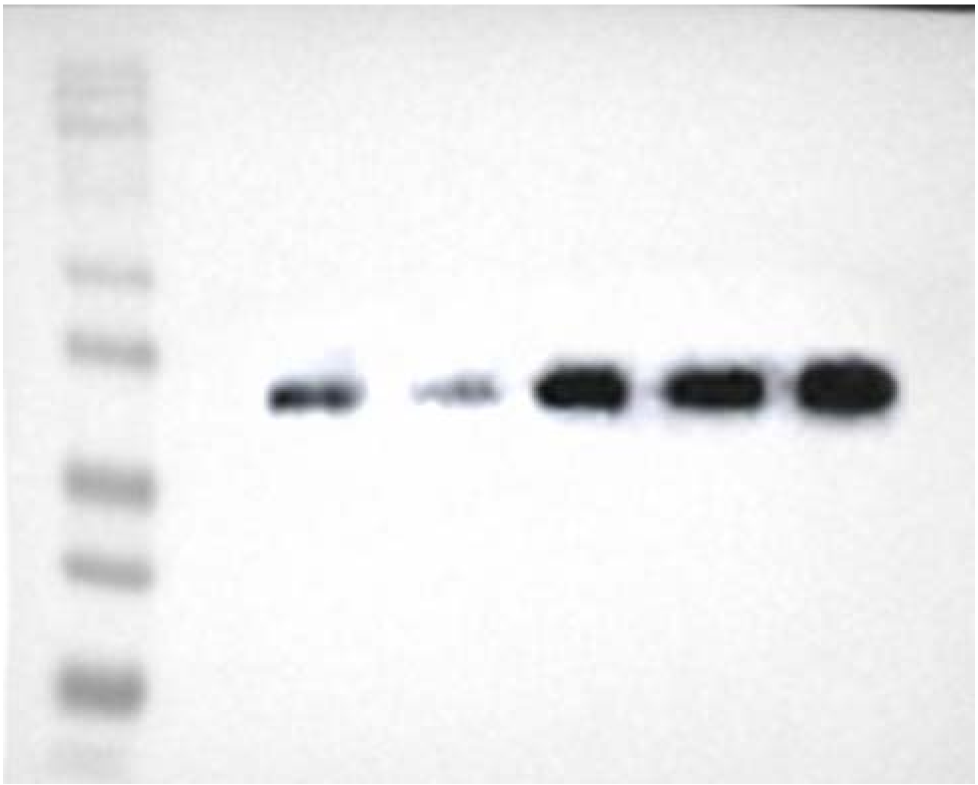

Figure5D Collagen-1

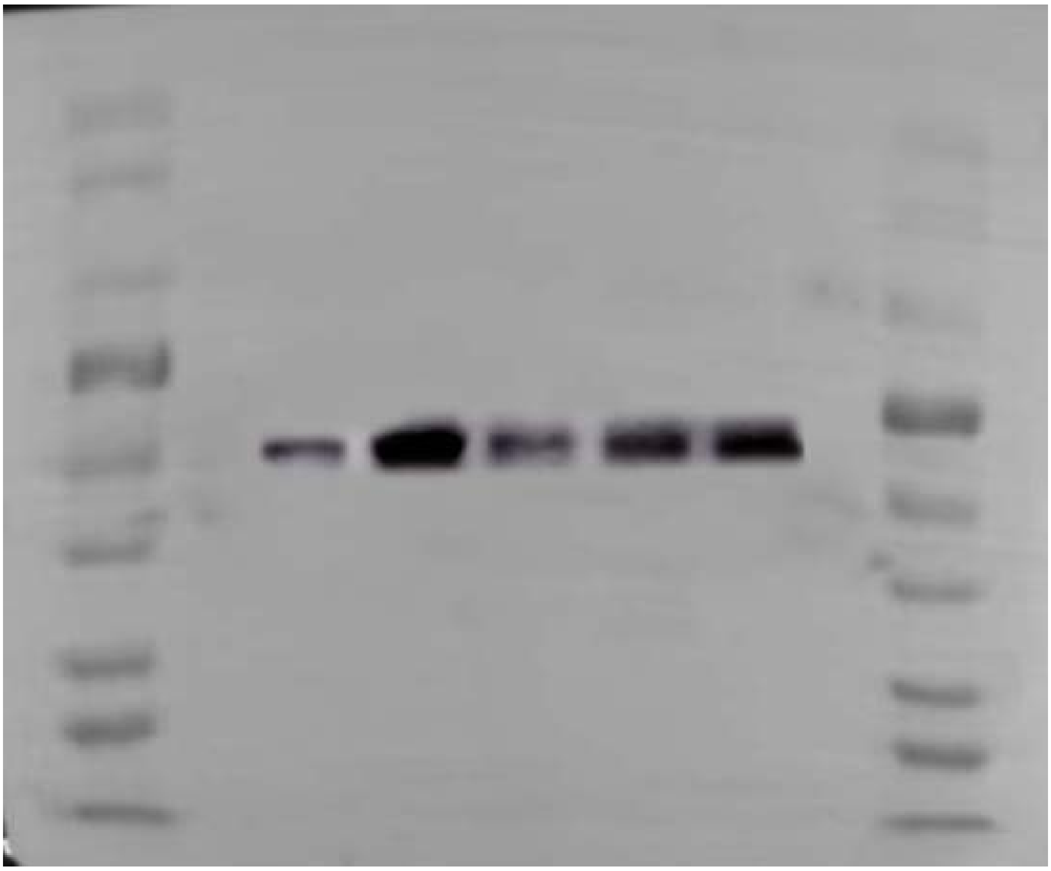

Figure5D Collagen-3

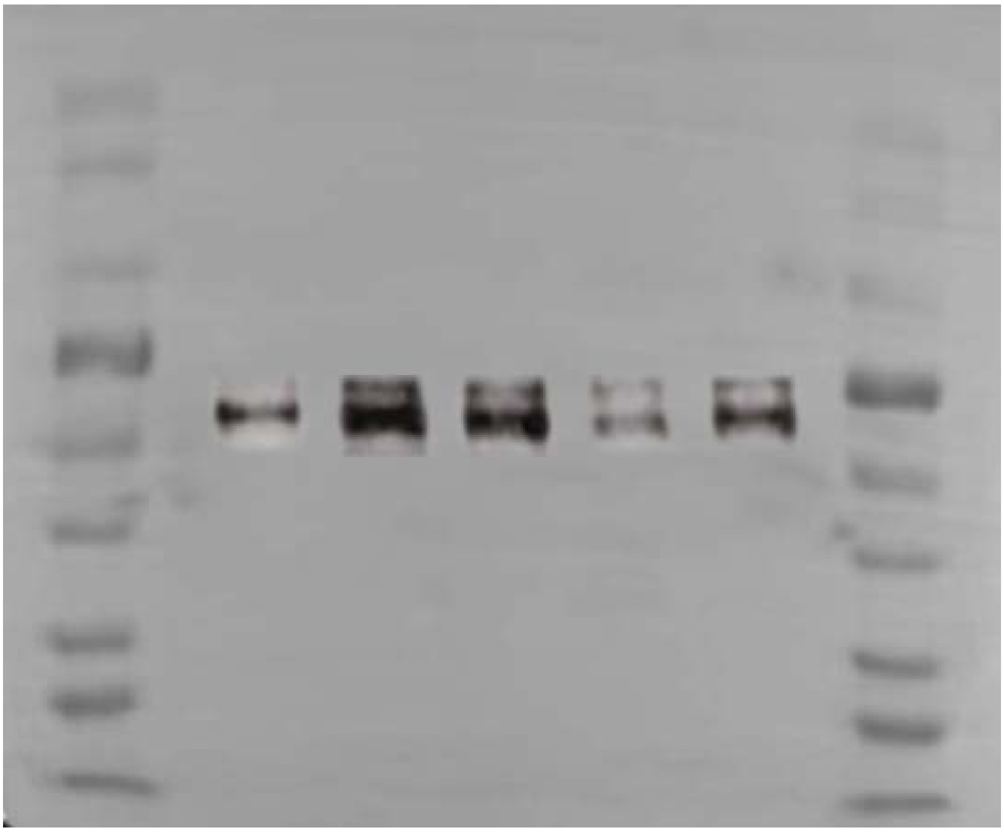

Figure5D CTGF

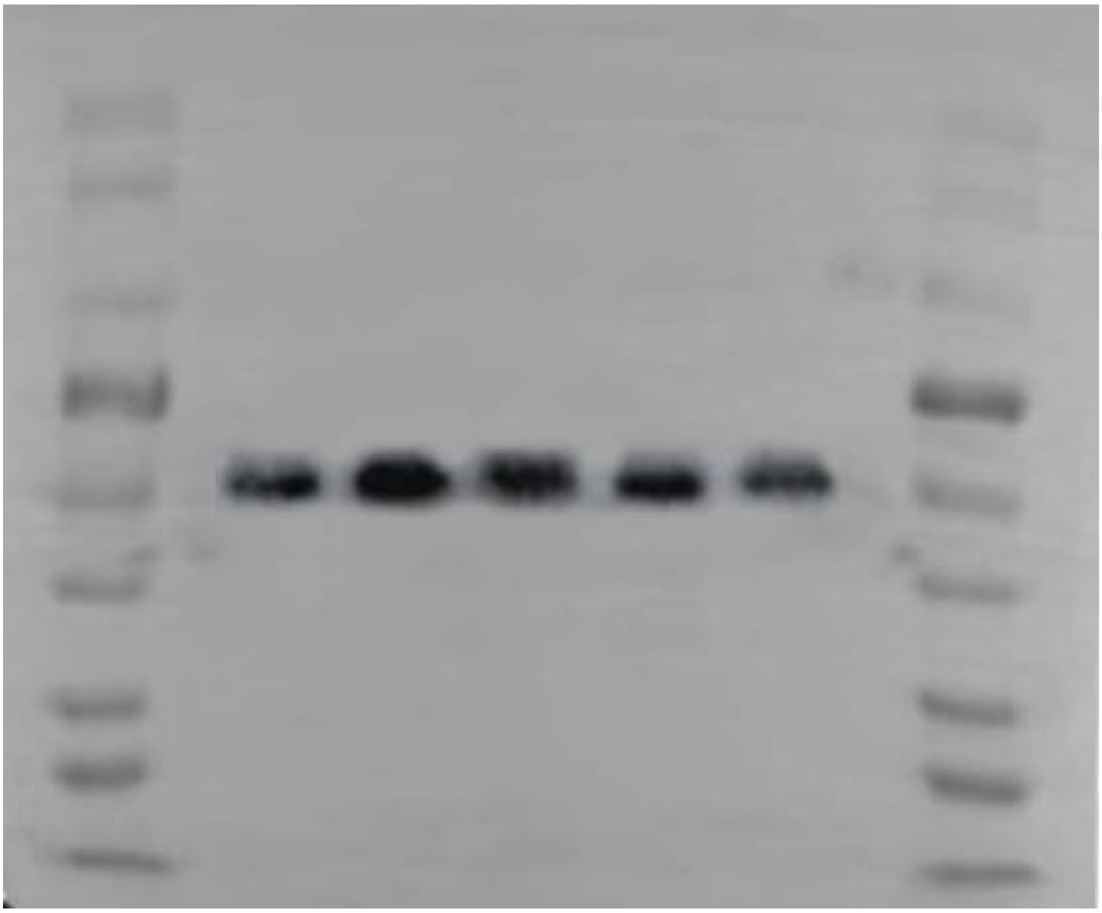

Figure5D MMP2

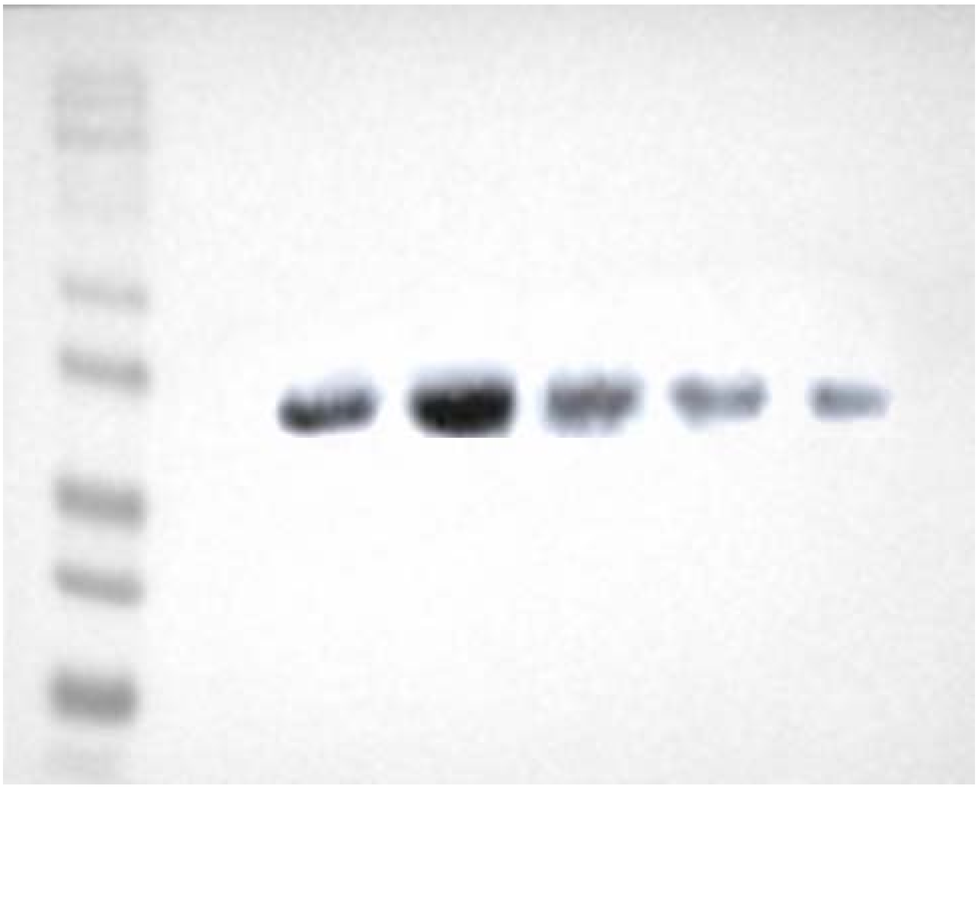

Figure5D MMP9

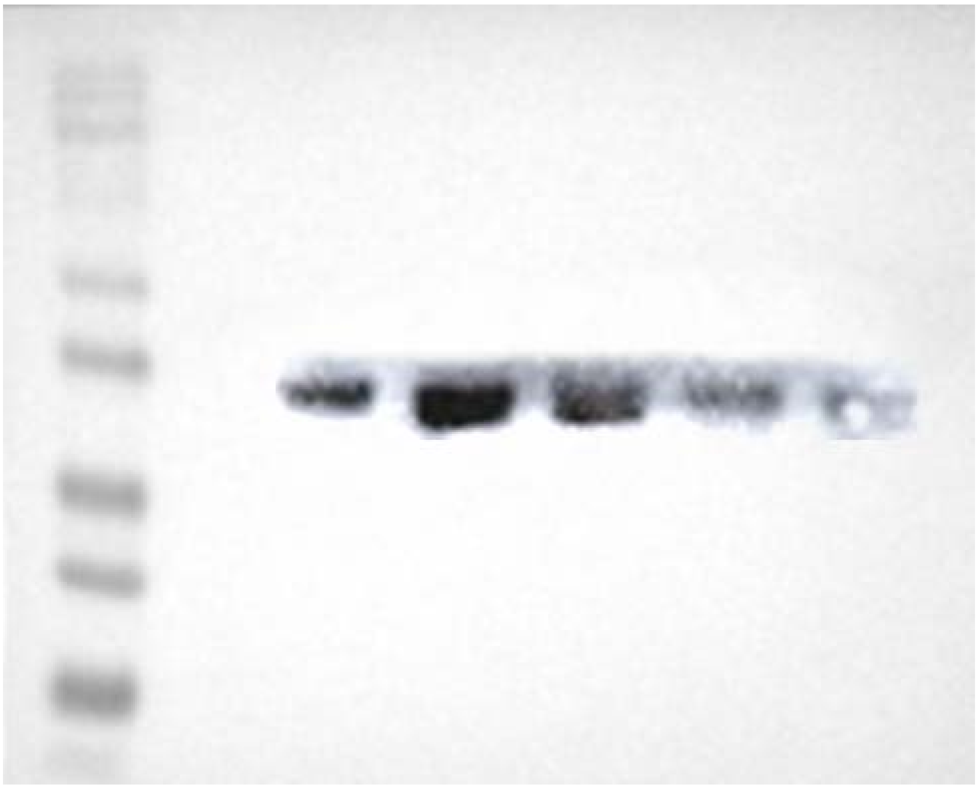

Figure5D α-SMA

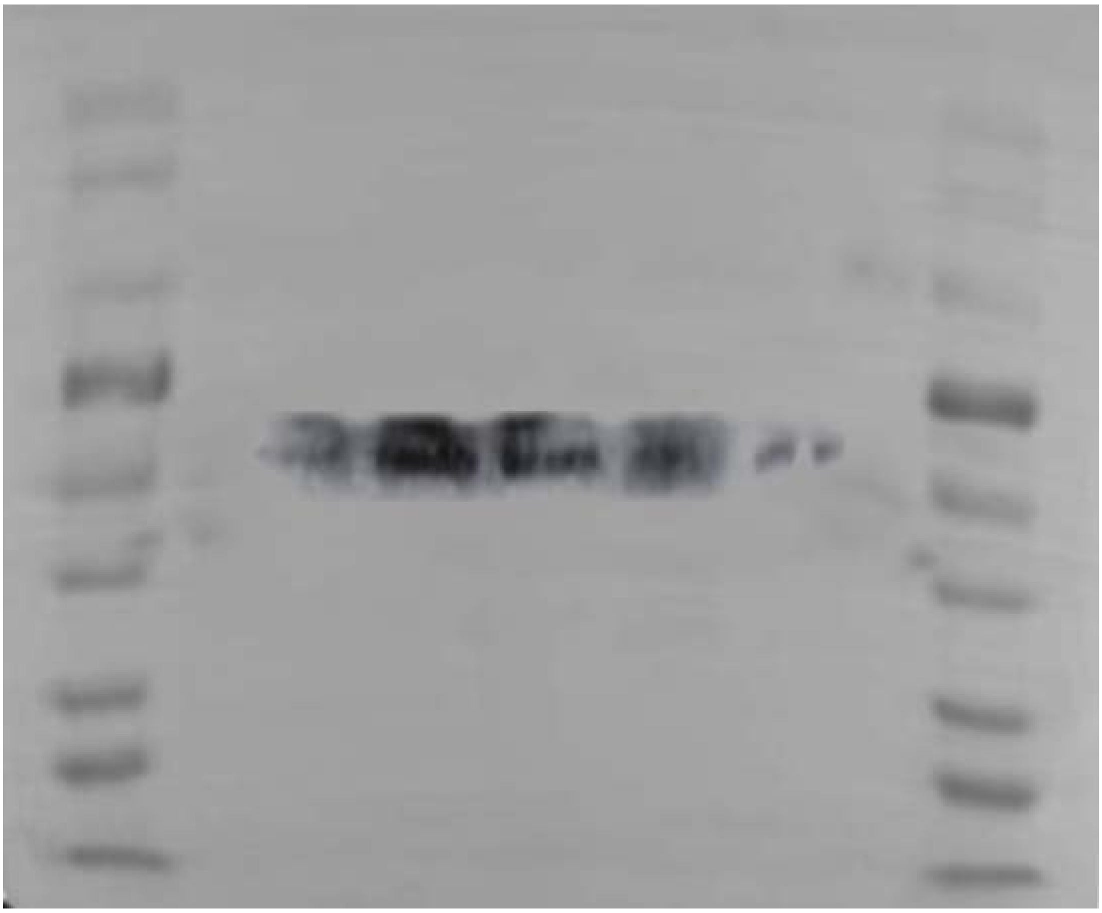

Figure5D β-actin

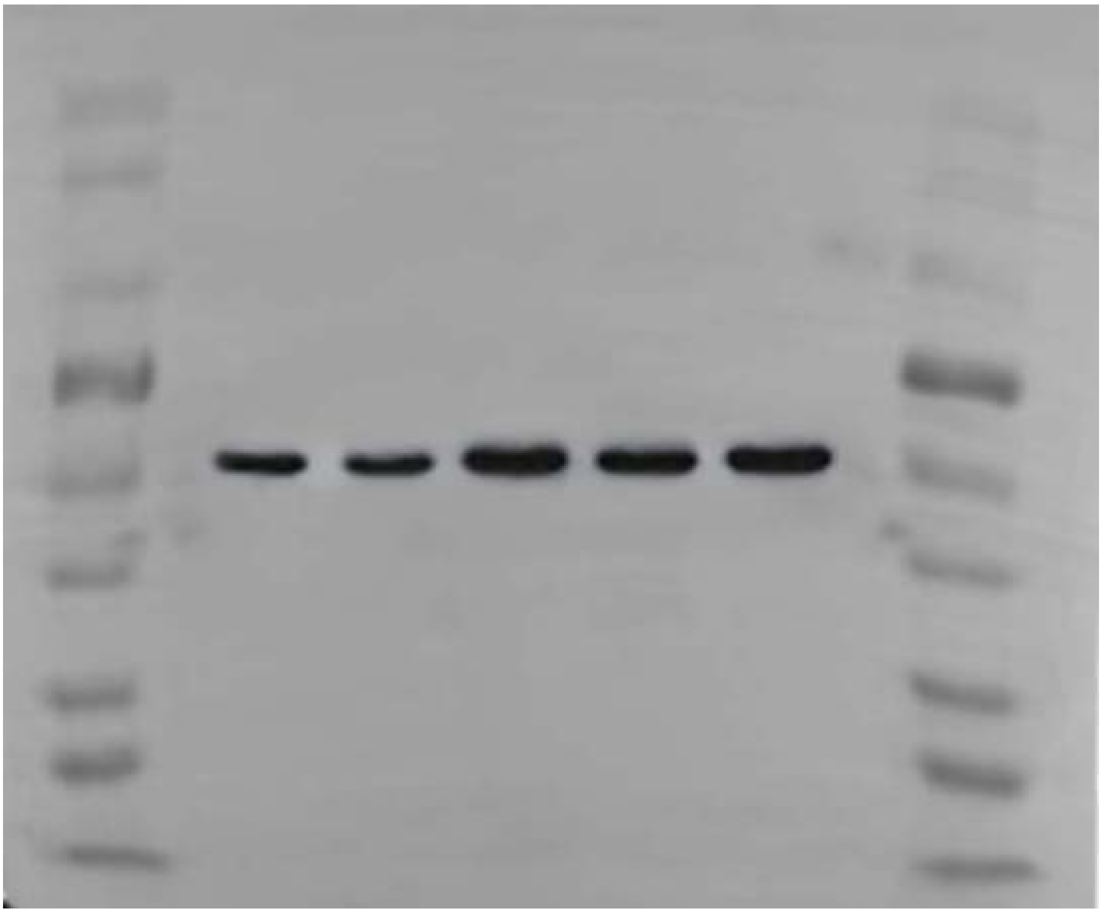

Figure5D1 GAPDH

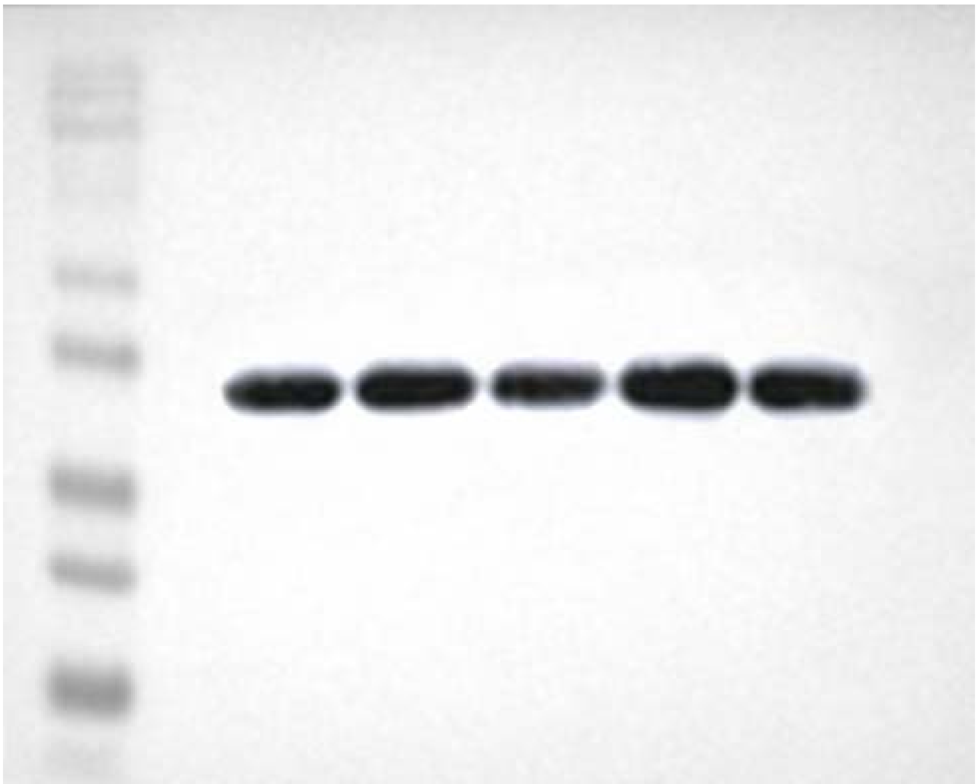

Figure5D2 GAPDH

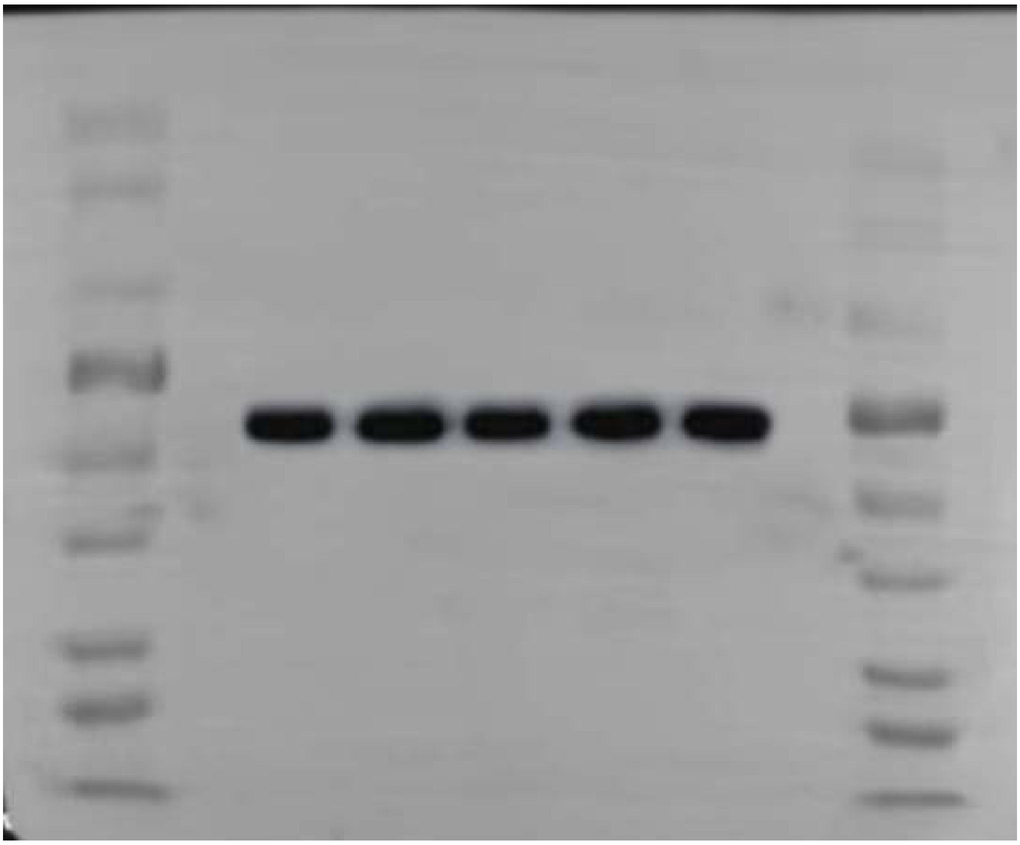

Figure5D3 GAPDH

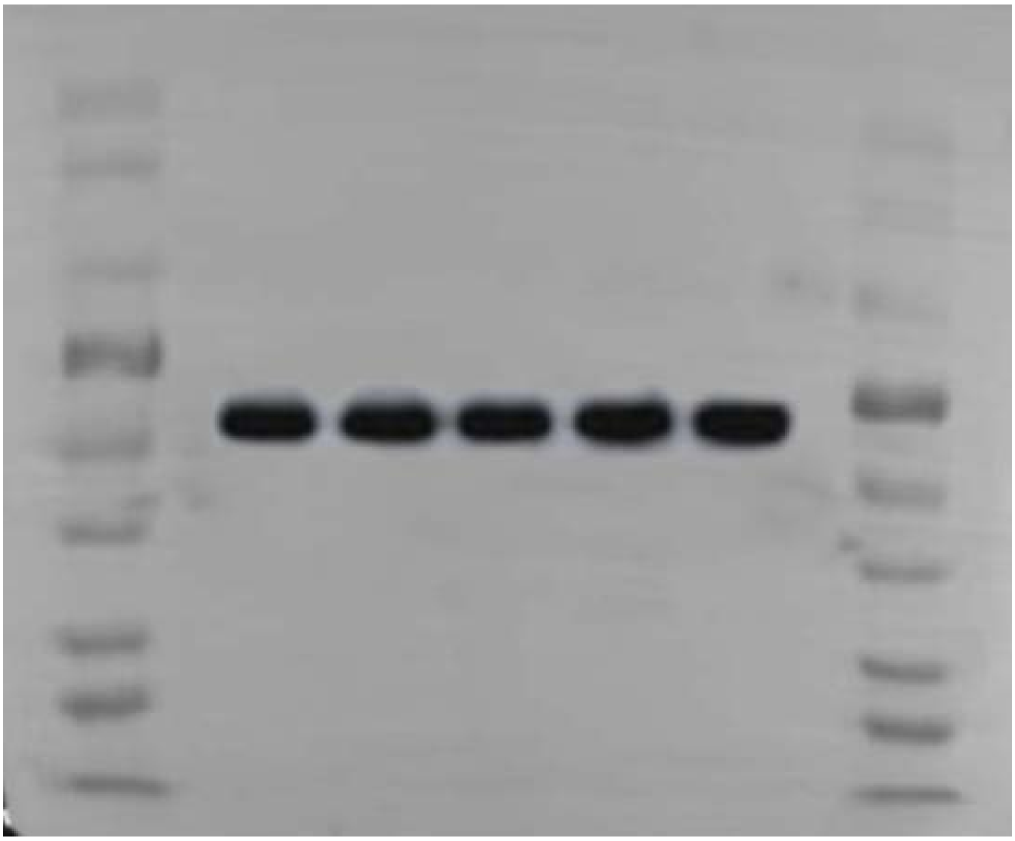

Figure5D4 GAPDH

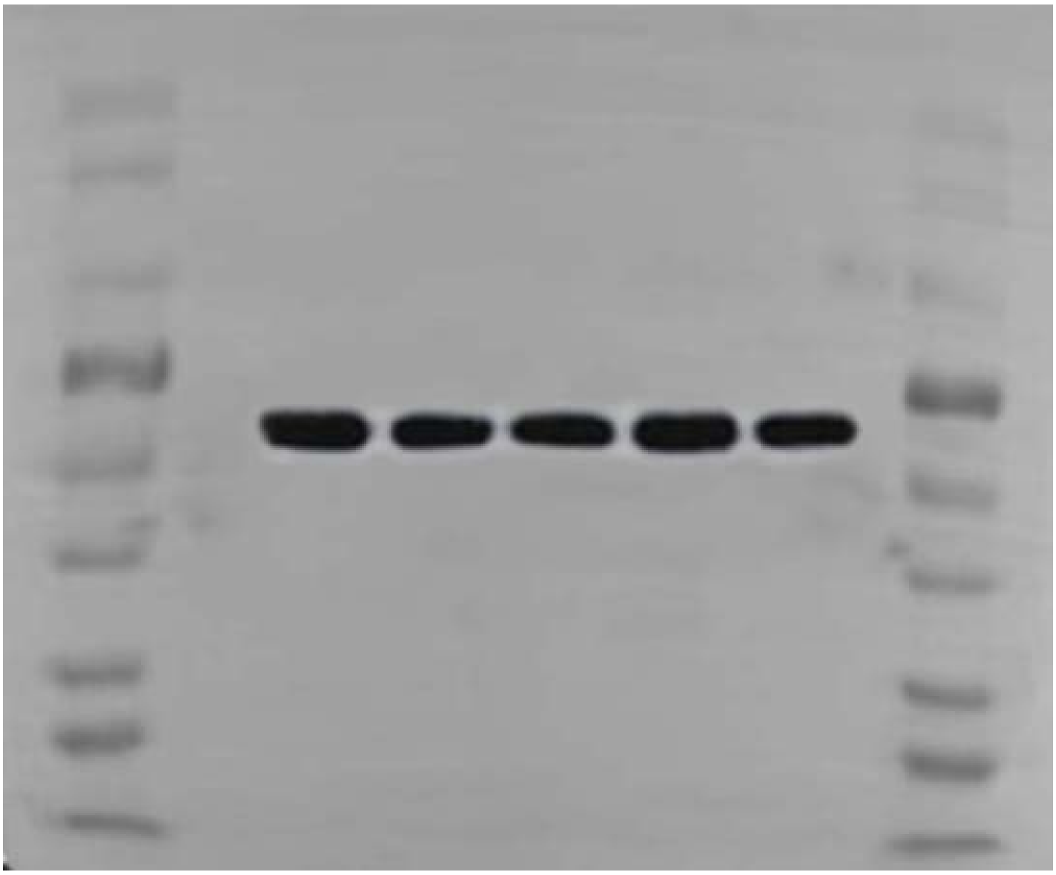

Figure5D5 GAPDH

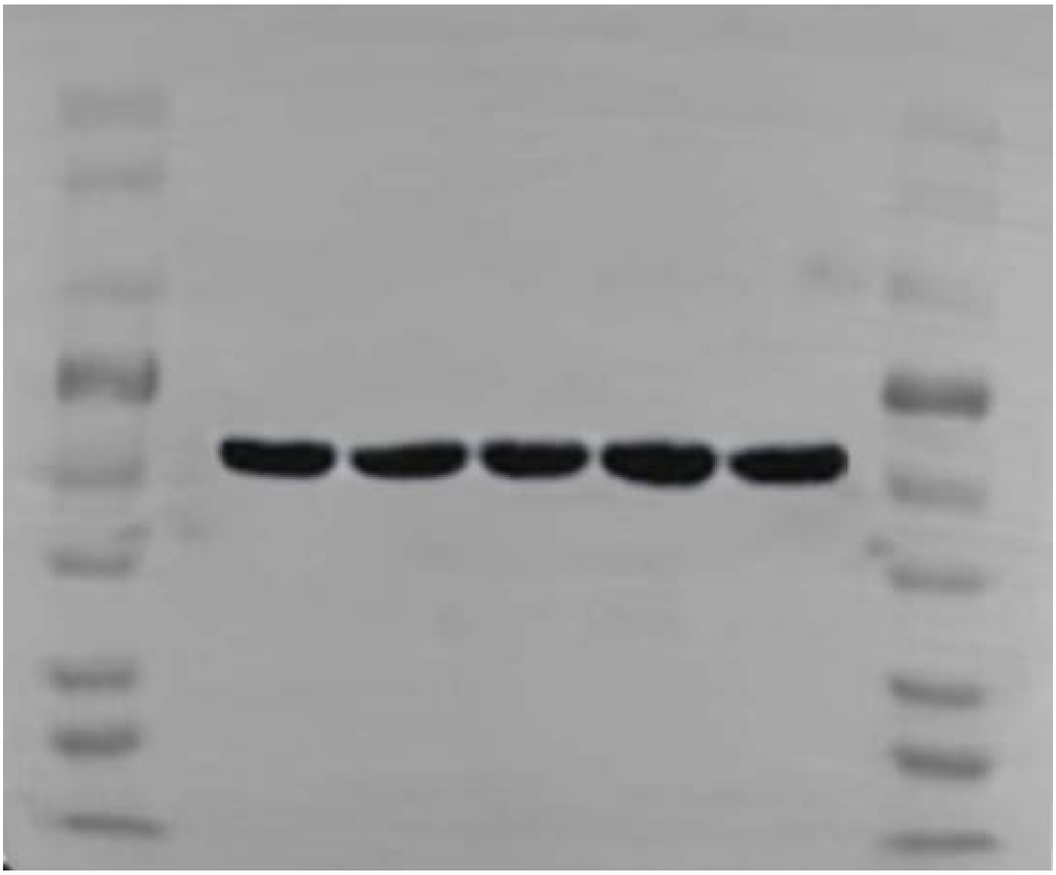

Figure6A p-Sam23

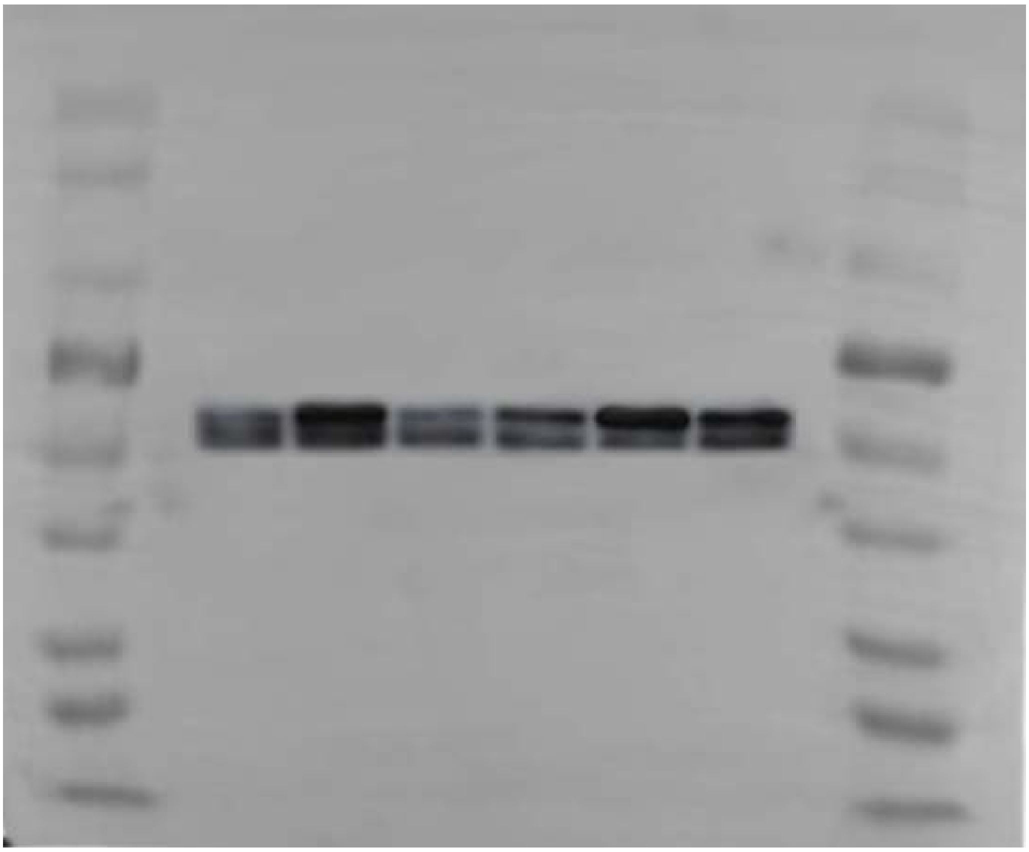

Figure6A Sam23

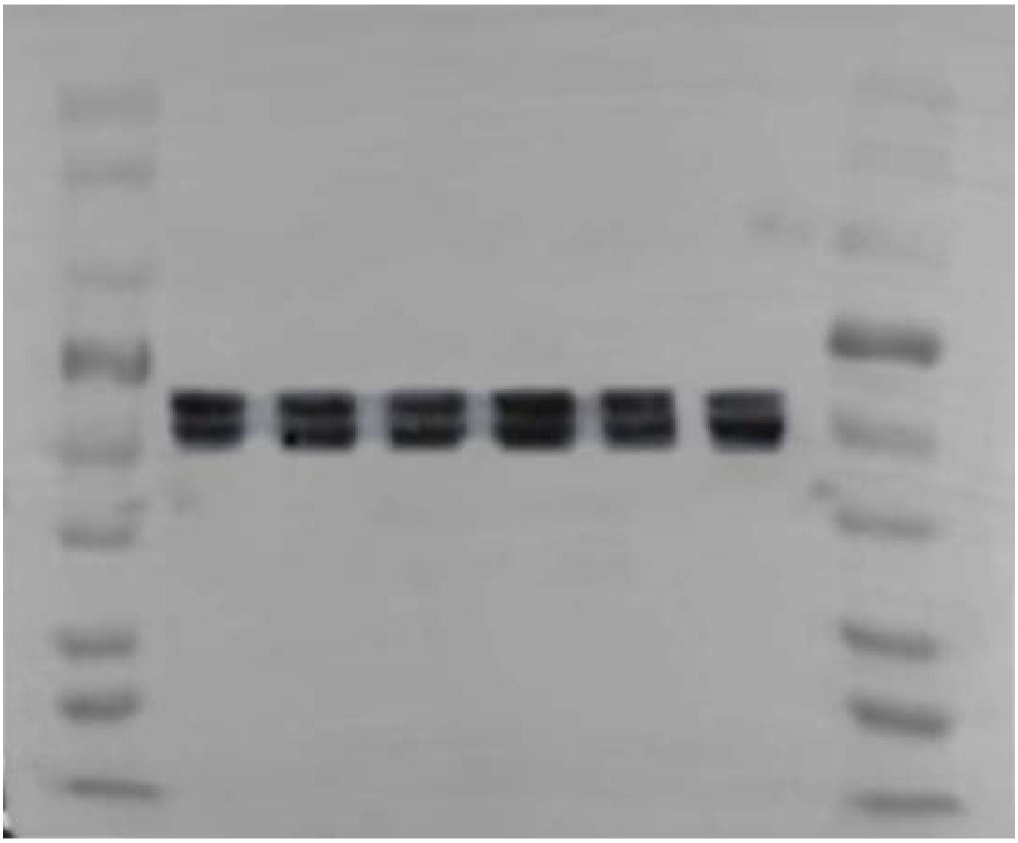

Figure6A TGF-β1

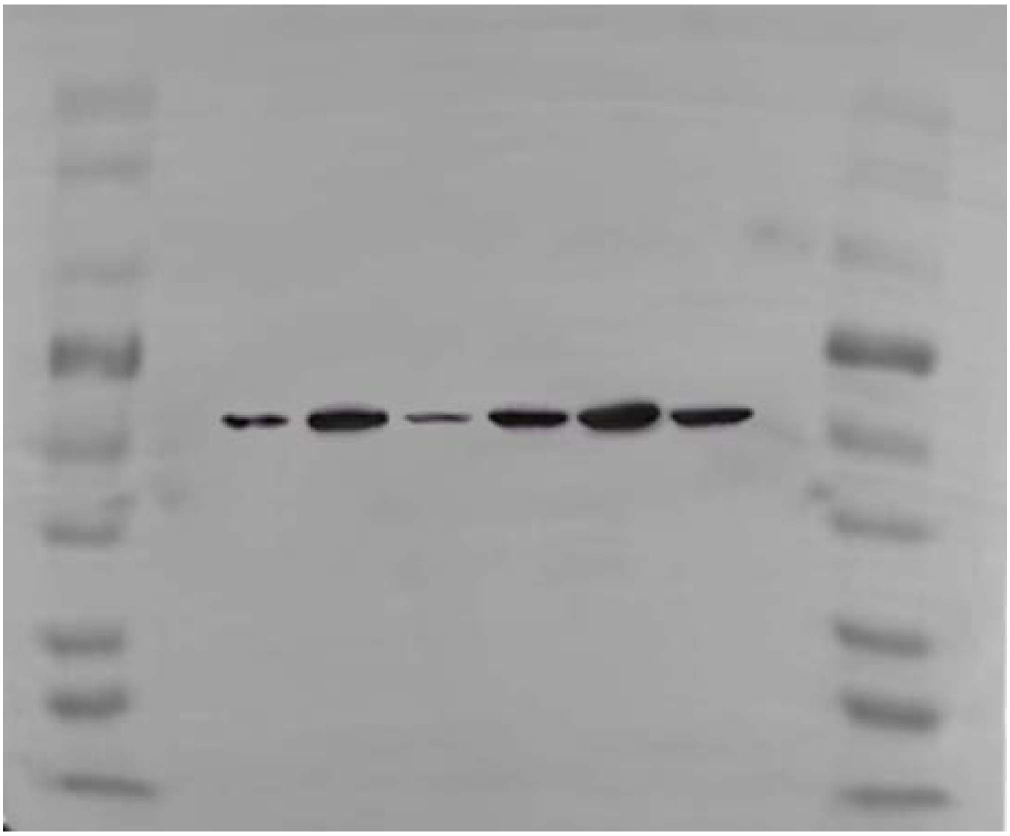

Figure6A1 GAPDH

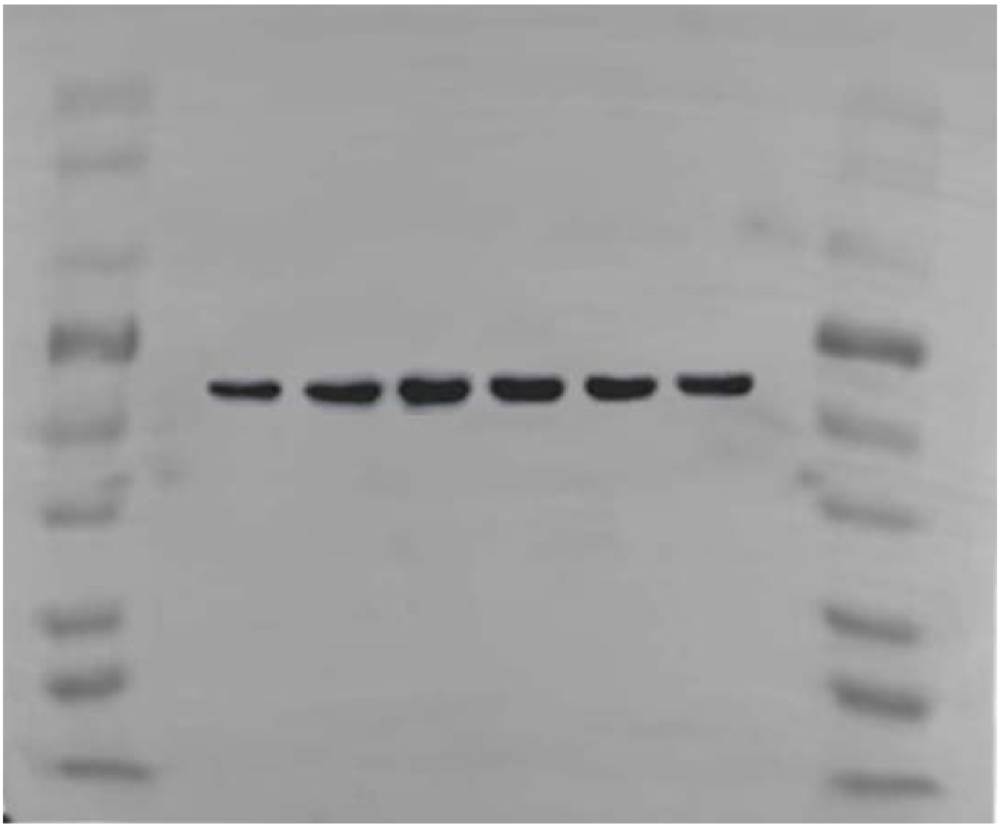

Figure6A2 GAPDH

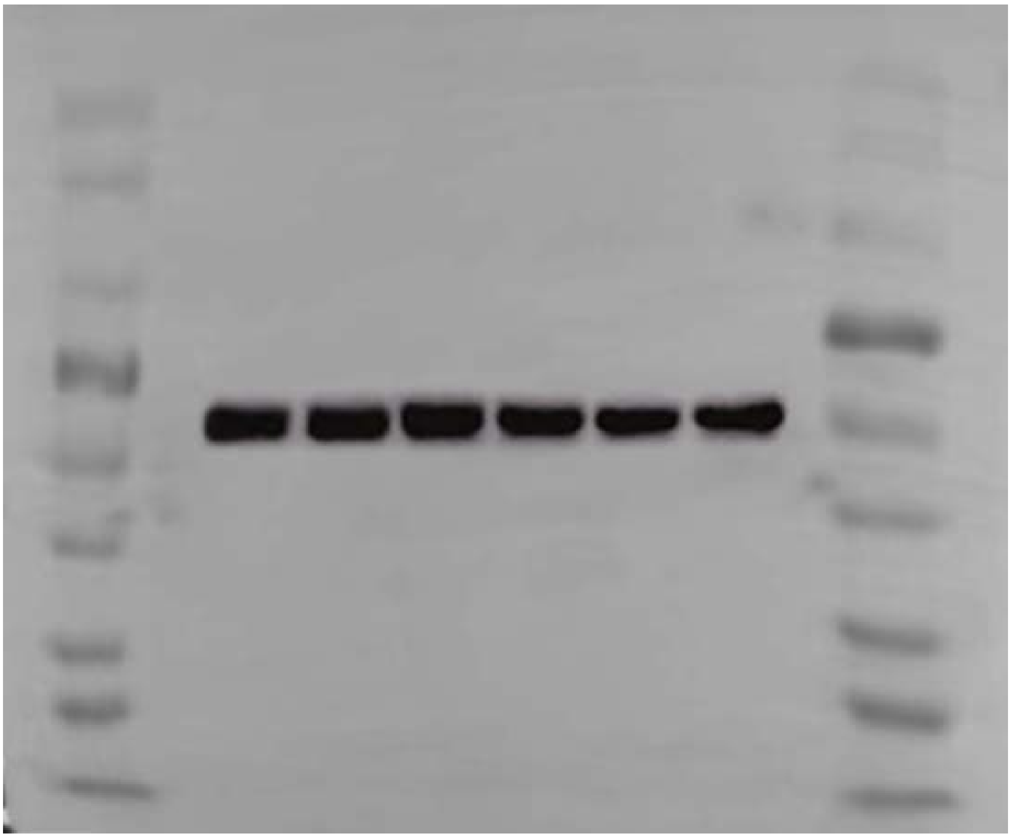

FigureF collagen-1

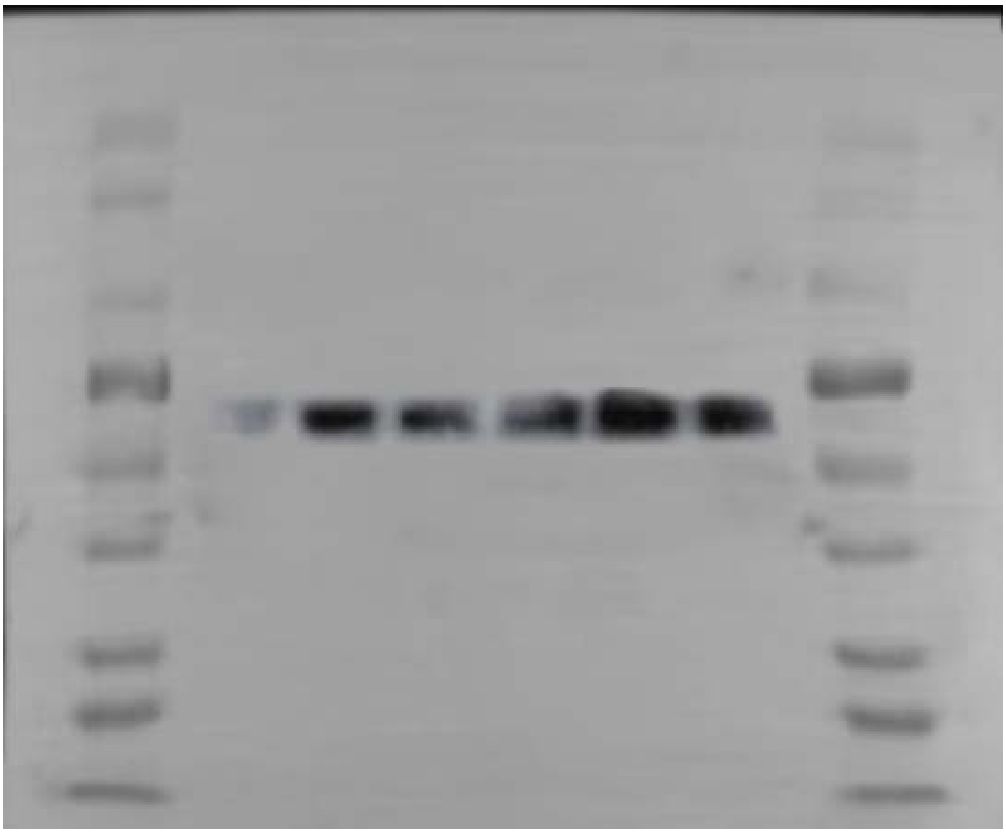

FigureF1 GAPDH

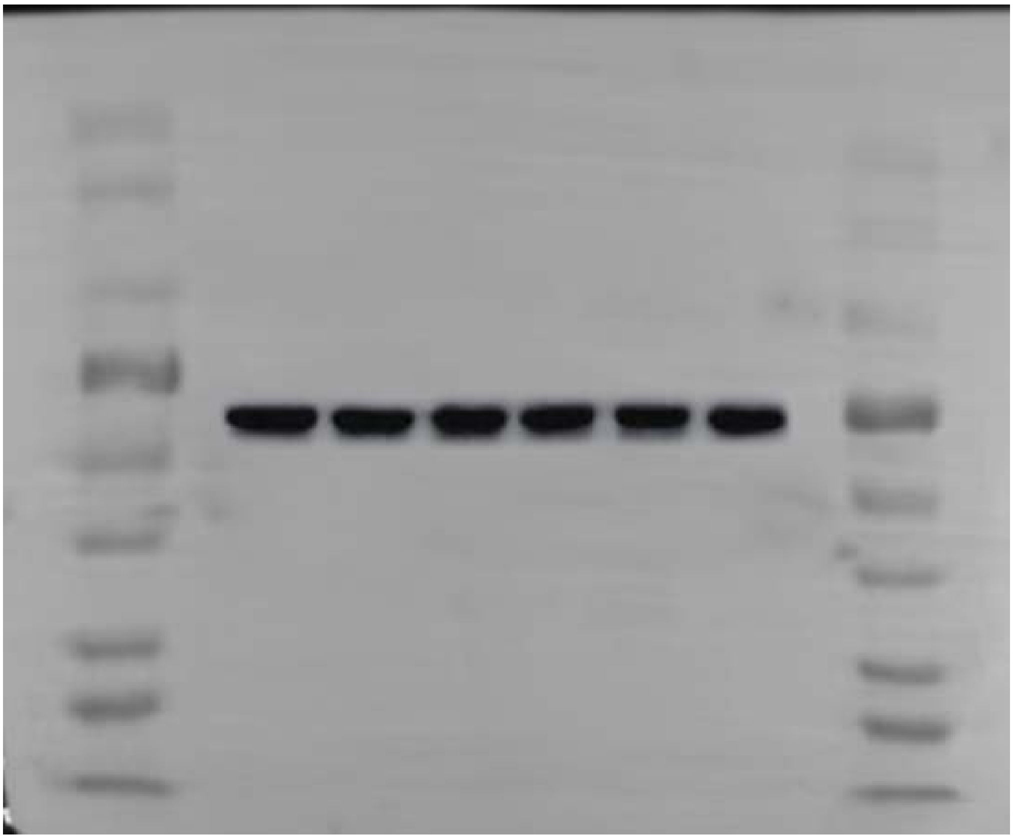

FigureF2 GAPDH

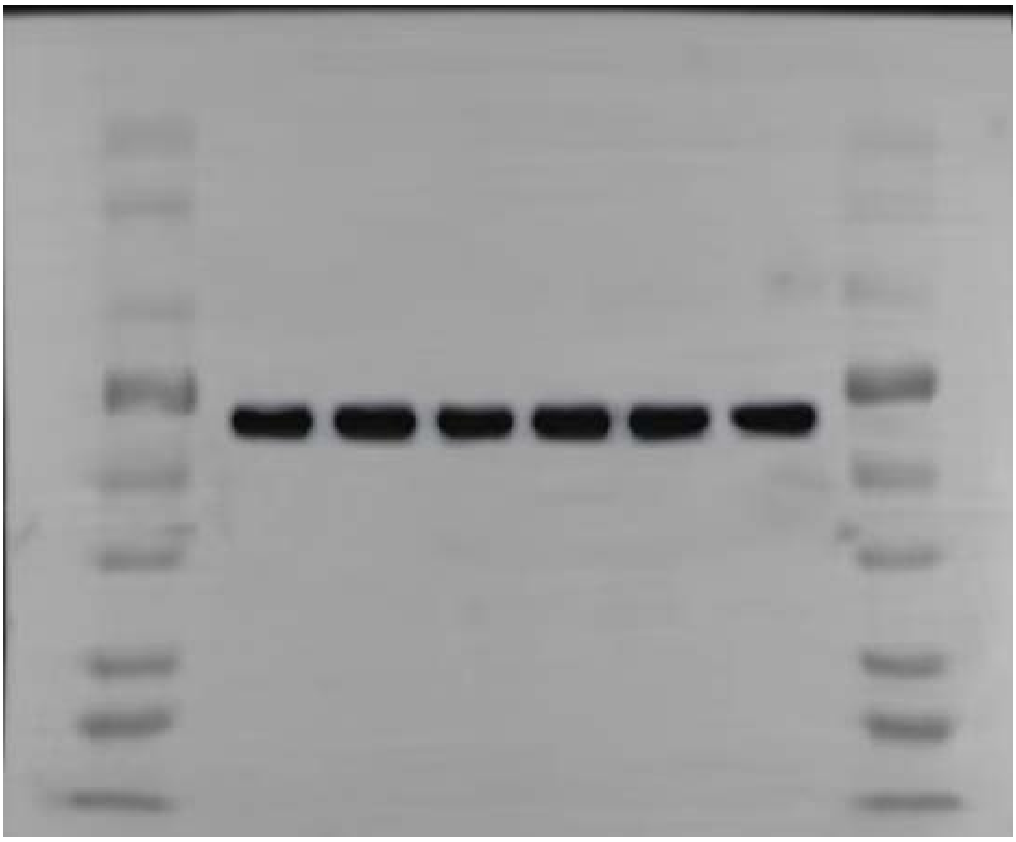

Supplement: S1 File — (PDF) [file pone.0342468.s001.pdf]
